# Somatic POLE exonuclease domain mutations are early events in sporadic endometrial and colorectal carcinogenesis, determining driver mutational landscape, clonal neoantigen burden and immune response

**DOI:** 10.1002/path.5081

**Published:** 2018-04-30

**Authors:** Daniel Temko, Inge C Van Gool, Emily Rayner, Mark Glaire, Seiko Makino, Matthew Brown, Laura Chegwidden, Claire Palles, Jeroen Depreeuw, Andrew Beggs, Chaido Stathopoulou, John Mason, Ann‐Marie Baker, Marc Williams, Vincenzo Cerundolo, Margarida Rei, Jenny C Taylor, Anna Schuh, Ahmed Ahmed, Frédéric Amant, Diether Lambrechts, Vincent THBM Smit, Tjalling Bosse, Trevor A Graham, David N Church, Ian Tomlinson

**Affiliations:** ^1^ Evolution and Cancer Laboratory, Barts Cancer Institute, Barts and the London School of Medicine and Dentistry Queen Mary University of London London UK; ^2^ Centre for Maths and Physics in the Life Sciences and Experimental Biology (CoMPLEX) University College London London UK; ^3^ Department of Computer Science University College London London UK; ^4^ Department of Pathology Leiden University Medical Centre Leiden The Netherlands; ^5^ Wellcome Trust Centre for Human Genetics University of Oxford Oxford UK; ^6^ KU Leuven (University of Leuven), University Hospitals Leuven, Department of Obstetrics and Gynaecology Division of Gynaecological Oncology Leuven Belgium; ^7^ KU Leuven, Department of Human Genetics Laboratory for Translational Genetics Leuven Belgium; ^8^ VIB Centre for Cancer Biology Laboratory for Translational Genetics Leuven Belgium; ^9^ Institute of Cancer and Genomic Sciences University of Birmingham Birmingham UK; ^10^ Department of Cell and Developmental Biology University College London London UK; ^11^ MRC Human Immunology Unit, Weatherall Institute of Molecular Medicine University of Oxford Oxford UK; ^12^ Department of Oncology University of Oxford Oxford UK; ^13^ Ovarian Cancer Cell Laboratory, Weatherall Institute of Molecular Medicine University of Oxford Oxford UK; ^14^ Nuffield Department of Obstetrics & Gynaecology University of Oxford Oxford UK; ^15^ Centre for Gynaecological Oncology Amsterdam Netherlands Cancer Institute Amsterdam The Netherlands

**Keywords:** POLE, polymerase proofreading, mutation, endometrial cancer, colorectal cancer, precursor lesion

## Abstract

Genomic instability, which is a hallmark of cancer, is generally thought to occur in the middle to late stages of tumourigenesis, following the acquisition of permissive molecular aberrations such as TP53 mutation or whole genome doubling. Tumours with somatic POLE exonuclease domain mutations are notable for their extreme genomic instability (their mutation burden is among the highest in human cancer), distinct mutational signature, lymphocytic infiltrate, and excellent prognosis. To what extent these characteristics are determined by the timing of POLE mutations in oncogenesis is unknown. Here, we have shown that pathogenic POLE mutations are detectable in non‐malignant precursors of endometrial and colorectal cancer. Using genome and exome sequencing, we found that multiple driver mutations in POLE‐mutant cancers show the characteristic POLE mutational signature, including those in genes conventionally regarded as initiators of tumourigenesis. In POLE‐mutant cancers, the proportion of monoclonal predicted neoantigens was similar to that in other cancers, but the absolute number was much greater. We also found that the prominent CD8^+^ T‐cell infiltrate present in POLE‐mutant cancers was evident in their precursor lesions. Collectively, these data indicate that somatic POLE mutations are early, quite possibly initiating, events in the endometrial and colorectal cancers in which they occur. The resulting early onset of genomic instability may account for the striking immune response and excellent prognosis of these tumours, as well as their early presentation. © 2018 The Authors. The *Journal of Pathology* published by John Wiley & Sons Ltd on behalf of Pathological Society of Great Britain and Ireland.

## Introduction

Next‐generation sequencing (NGS) technologies have hugely advanced our understanding of the mechanisms of tumourigenesis. The ability to analyse the entire genome or exome at depth in large numbers of tumours has substantially increased the list of driver genes – i.e. those that, when mutated, promote tumour growth. It has also revealed that such driver mutations are not always present in the dominant tumour clone 1, 2. This is clinically relevant, because targeting of subclonal drivers is likely to kill only a subpopulation of tumour cells, whereas successful targeting of clonal variants may lead to tumour eradication. Thus, differentiating early, clonal mutations from late, subclonal ones may not only increase our understanding of the mechanisms of oncogenesis, but also inform the clinical management of patients 2.

Fundamentally, all mutations are caused, in part, by a failure to recognize or repair defects in DNA sequence or chromosome structure. In many cancers, this is a consequence of specific defects in the cellular processes responsible for maintaining genomic integrity 3. One recently described example is the genomic instability caused by missense mutations in the exonuclease (proofreading) domains of the major replicative DNA polymerase genes POLE and POLD1 4. Polymerase proofreading recognizes and corrects mispaired bases incorporated during DNA replication; its perturbation as a result of these mutations is associated with an exceptional number of single‐nucleotide variants (SNVs) (although not indels), and a distinct mutational signature typified by C:G → A:T transversions, in which the mutated cytosine is in the context TCT, and C:G → T:A transitions, in which the mutated cytosine is in the context TCG 4, 5, 6. POLE and POLD1 exonuclease domain mutations may occur in the germline, where they cause polymerase proofreading‐associated polyposis – a condition characterized by intestinal polyposis and tumours of the colorectum and uterus, among other organs 7. Somatic POLE exonuclease domain mutations (hereafter simply referred to as POLE mutations) occur in sporadic tumours of the endometrium (7–15% cases) 8, 9, colorectum (1–2%) 10, 11, and, less commonly, in other cancers (although, for reasons that are unclear, somatic POLD1 exonuclease domain mutations are very uncommon). POLE‐mutant colorectal and endometrial cancers have an excellent prognosis 8, 11, 12, 13, probably owing to a robust antitumour immune response against the multitude of immunogenic neoantigens that they are predicted to harbour 11, 14, 15. Very recent reports have also suggested that these tumours may be highly responsive to immune checkpoint inhibition 16.

Although it is clear that somatic POLE mutation causes a mutator phenotype 17 and acts as a cancer driver 4, 5, several questions about its contribution to tumourigenesis remain unanswered. One of the most important of these relates to the timing of these mutations in cancer development. If POLE mutations are late events, their consequences may be restricted to a subclone of tumour cells, the targeting of which may fail to meaningfully alter tumour behaviour. In contrast, if POLE mutations occur early, they could rapidly cause a large number of clonal alterations that may alter prognosis or response to therapy. This is particularly pertinent in the light of recent data suggesting that long‐term benefit from immune checkpoint inhibition is limited to patients whose cancers harbour neoantigens in the dominant tumour clone 18. In contrast to germline mutations in DNA repair pathways in rare inherited syndromes (such as the mismatch repair gene variants that cause Lynch syndrome), the acquisition of genomic instability in sporadic cancers has largely been believed to be a mid‐stage to late‐stage event during carcinogenesis 19. For example, in sporadic colorectal cancer – a tumour type in which the molecular progression of precancers (adenomas) to invasive carcinomas has been well characterized – mismatch repair deficiency (MMR‐D) or chromosomal instability occur after initiating (epi)mutations in APC, BRAF, or KRAS, or other events such as whole genome doubling or loss of chromosome 18q 19, 20, 21, 22, 23, 24. Thus, in addition to its clinical relevance, the demonstration that the POLE mutator phenotype operates from the first stages of tumour initiation would also reveal a novel pathway of sporadic tumourigenesis. A recent case report of a pathogenic POLE mutation in a endometrial cancer and its precursor 25 suggests that these mutations may occur early in tumour development, but the single case precludes generalization of this result.

In this study, we comprehensively examined the timing of pathogenic somatic POLE mutations in sporadic endometrial and colorectal cancers by using tumour whole genome sequencing (WGS), public sequencing data from The Cancer Genome Atlas (TCGA) 8, 10, and targeted sequencing of additional cohorts of cancers and precancers.

## Materials and methods

### Ethical approval

Patient consent for research on tumour tissue was obtained at the recruiting centres under local ethical approval. Molecular analysis of anonymized tissue was performed under Oxford Research Ethics Committee A approval (05/Q1605/66).

### Patients and tumour samples

Details of the cohorts and cases analysed in this study are shown in supplementary material, Tables S1 and S2. Fifty‐one formalin‐fixed paraffin‐embedded (FFPE) endometrial cancers carrying known pathogenic somatic POLE mutations identified in our previous studies 12, 14, 26 were reviewed for the presence of a concomitant and spatially discrete area of endometrial intraepithelial neoplasia (EIN) by examination of haematoxylin and eosin (H&E)‐stained slides by two expert gynaecological pathologists (V.S. and T.B.). An additional 389 FFPE colorectal polyps (tubular adenomas, tubulovillous adenomas, and serrated adenomas – hereafter referred to as adenomas), for which POLE screening had not previously been performed, were identified from 261 participants in the CORGI study, which recruited patients with a family history of colorectal cancer and a personal history of a colorectal polyp or colorectal malignancy in the absence of a known tumour predisposition syndrome. Six fresh frozen tumours with pathogenic somatic POLE mutations (five endometrial; one colorectal) were identified from a Leuven endometrial cancer cohort used in our previous study 12, a prospective clinical sequencing programme (HICF2) at the University of Oxford, or the University of Birmingham tissue bank. TCGA colorectal (COADREAD) 10 and endometrial (uterine corpus endometrial carcinoma) 8 cancer data were downloaded from the Genomic Data Commons (GDC) Data Portal (https://portal.gdc.cancer.gov; June 2017). An additional series of 78 FFPE endometrial cancers, including 32 cases with pathogenic somatic POLE mutations, were identified from the Leiden University Medical Centre (LUMC) archives (2001–2015) 14. Further details of the cohorts used in this study are provided in supplementary material, Table S1. Molecular analyses were performed on a single tumour or precursor lesion region in each case.

### DNA extraction

After review to confirm adequate tumour cellularity, DNA was extracted from fresh frozen or microdissected FFPE tumours and precursors with standard methods [Roche FFPE‐T DNA kit (F. Hoffman La Roche AG, Basel, Switzerland), Machery Nagel Nucleospin DNA FFPE XS (Machery Nagel, Duren, Germany)/FFPE DNA kit, or Qiagen Blood and Tissue kit (Qiagen, Hilden, Germany)] and resuspended in buffer or water.

### DNA sequencing

Full details of the sample preparation and the sequencing methods utilized in this study are provided in supplementary material, Supplementary materials and methods. In brief, EINs and paired carcinomas were sequenced for mutations in 30 cancer genes by the use of molecular inversion probe capture, and a custom version of the 72‐gene Ion AmpliSeq Cancer Hotspot panel v2 (including 80 genes; ThermoFisher, Waltham, MA, USA) (supplementary material, Tables S3 and S4). WGS of fresh frozen tumours was performed with Illumina HiSeq (Illumina, San Diego, CA, USA), and aligned to the reference genome with BWA mem or Isaac 27. FFPE endometrial cancers from the LUMC series were analysed by use of the Lifetech/ThermoFisher Ion AmpliSeq Comprehensive Cancer Panel comprising 409 cancer genes (http://www.lifetechnologies.com/order/catalog/product/4477685). Mutation calling was performed with LoFreq 28 (EINs), Mutect, Mutect2 29, or Strelka 30 (WGS and TCGA cases), or Ion Torrent variantCaller (EINs and LUMC FFPE tumours). Copy number profiles were derived with Sequenza 31. Variant annotation was performed with using Annovar 32 or Variant Effect Predictor 33.

### Definition of driver genes

Driver genes were defined according to the IntOGen driver gene repository (https://www.intogen.org/search), and included both PanCancer (Pooled_driver) and tumour type‐specific (perProject_driver) variants (supplementary material, Tables S5 and S6) 34. High‐confidence driver mutations (defined as either truncating mutations in genes likely to be tumour suppressors, or recurrent missense mutations in any endometrial or colorectal cancer‐specific or pan‐cancer gene from the IntOGen set) were determined for a subset of driver genes by manual curation, blinded to tumour molecular characteristics.

### Clonality of POLE mutations

Most (36 of 38) endometrial and colorectal cancers with pathogenic POLE mutations were disomic at the POLE locus (chromosome 12q24) and were informative for clonality analysis. Of these, 20 of 22 endometrial cancers and 12 of 14 colorectal cancers had available copy number annotation. As all 32 of these showed near‐diploid genomes (>80% of the genome), we assumed diploid genomes for the four remaining cases.

Mutations were filtered to include only autosomal variants in diploid regions of the genome, called with a depth of at least 20×. Mutation allele frequency distributions were generated with the R ‘histogram’ function, and tumour cellularity was inferred as twice the midpoint of the allele frequency bin with the highest mutation density, excluding bins with a lower bound below an allele frequency of 0.1. These values were then subjected to manual curation. The hypothesis that the mutation was present in every tumour cell was tested with a one‐sided binomial test, based on the numbers of reference and variant reads at the POLE mutation site and the inferred tumour cellularity. Specifically, for a mutation with coverage R, in a tumour with tumour cell fraction C, the number of variant reads was modelled as a random variable X, with the distribution:

$$X∼\text{Binom}\left(R,C/2\right)$$

In each case, we calculated the probability, p, of finding the observed number of variant reads, v, or fewer, P(X ≤ v). Mutations were considered to be subclonal for p ≤ 0.05.

### Mutational signatures

Previously reported mutational signatures were obtained from http://cancer.sanger.ac.uk/cosmic/signatures/ on 1 June 2017. The complement of mutational processes active in the life‐history of each tumour sample was inferred by classification of mutations into 96 categories following Alexandrov 6, and the use of non‐negative least squares regression, implemented in the R package ‘nnls’. For this analysis, only mutational signatures previously reported as active in that cancer type (endometrial signatures 1, 2, 5, 6, 10, 13, 14, and 26; colorectal signatures 1, 5, 6, and 10) were used for the regression. For cases analysed by whole exome sequencing (WES), mutational signatures were re‐scaled to exomic trinucleotide frequencies. A mutational process was deemed to have been active in the life‐history of a tumour if the associated mutational signature had a coefficient of at least 2% of the total coefficients in the best‐fitting model. Mutations likely to be due to POLE mutation were identified by considering mutational signatures as multinomial probability distributions caused by specific mutational processes. The probability of each mutation under all mutational processes active in that tumour was calculated, and mutations were assigned to the ‘POLE’ mutational process in cases where the probability under that process was at least twice the probability under any other process.

### POLE consensus mutational signature scores in driver genes

Tumour mutations were obtained from calling based on tumour/normal .bam files (POLE‐mutant cases) or TCGA MAF files [mismatch repair‐proficient (MMR‐P) and MMR‐D cases], and classified into 96 categories following Alexandrov 6. For each tumour, the distribution of mutations across the 96 types was calculated, and re‐scaled to equal trinucleotide frequencies based on sequencing type, providing an individual tumour mutational signature. Tumours were then categorized into three groups according to POLE mutation and mismatch repair status (i.e. POLE‐mutant, MMR‐P, and MMR‐D), and a consensus mutational signature was calculated for each group as the average of the individual‐tumour signatures among samples in the group, weighted by the number of mutations in each sample. The probability of all non‐silent mutations (‘non‐synonymous SNV’ or ‘stopgain’) in driver genes (as defined above) under each of the three consensus mutational signatures was then calculated, and the ratio of the probability of each mutation under the POLE consensus mutational signature compared to that under each of the other two consensus mutational signatures was obtained. For each individual gene, a ‘POLE score’ was then calculated as the base 2 logarithm of the minimum value of these ratios across all the non‐silent mutations within that gene.

### Immunohistochemistry

Immunohistochemistry (IHC) for CD8 was performed as reported previously 14. The number of CD8^+^ cells was quantified for the epithelial and stromal regions of the EIN. For the final CD8 count per case, the mean of these regions in 10 high‐power fields (HPFs) (625 × 425 μm) was calculated. A similar method was used to quantify CD8 density in colorectal adenomas, although the small lesion size meant that estimates were obtained from the mean of two or three HPFs.

### Clonal neoantigen prediction

We estimated the number of clonal neoantigens by using a modification of our previously reported algorithm 11, modified to predict peptide binding to patient‐specific human leukocyte antigen (HLA) molecules (determined from WGS or WES data with OptiType 35). Neoantigens were defined as mutations predicted to specify peptides that bound patient HLA molecules with an affinity of <500 nm. Copy number information was obtained from the GDC data portal, as described above. Clonality was determined as described above. Neoantigens were considered to be clonal if the binomial test P value was >0.05.

### Statistical analysis

Analyses were performed with R (CRAN network) or Prism (GraphPad Software, La Jolla, CA, USA). Statistical comparison between groups was performed with the non‐parametric Mann–Whitney U‐test. All P values were two‐sided, unless otherwise specified. Statistical significance was accepted at p < 0.05.

## Results

### Somatic POLE mutations are detectable in sporadic endometrial and colorectal precancers

**Figure 1:**
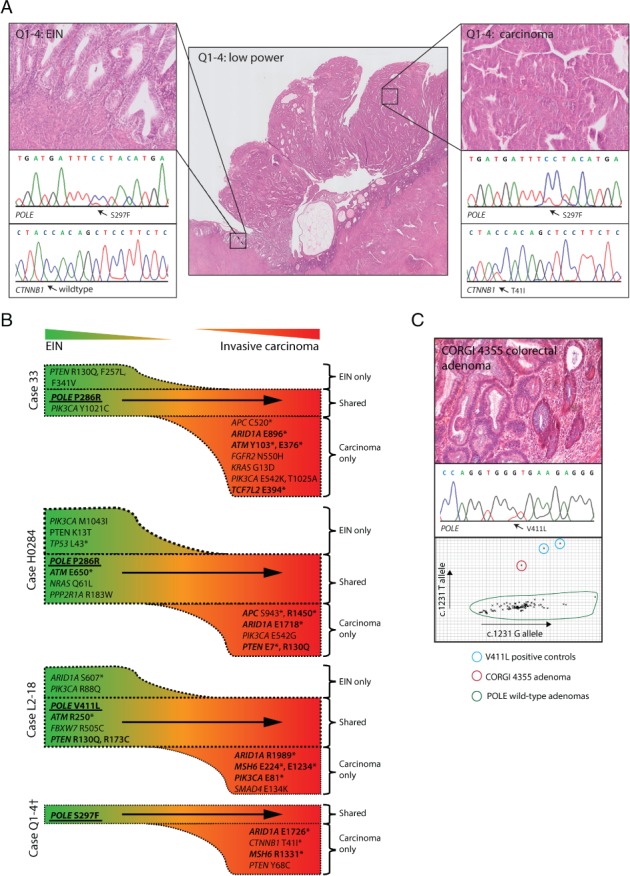
Pathogenic, somatic POLE mutations in precursors of endometrial and colorectal cancers. Expert histopathological review of 51 endometrial cancers with pathogenic POLE mutations revealed four with concomitant and spatially discrete areas of EIN. (A) H&E‐stained section from one case with the results of Sanger sequencing of the malignant and precursor components. (B) Targeted sequencing of paired endometrial lesions by the use of two orthogonal NGS panels revealed that POLE mutations (bold, underlined) were present in both EIN and carcinomas in all cases (validated by Sanger sequencing in all cases). In each case, progression of EIN to endometrial carcinoma was associated with the gain of driver mutations, several of which were glutamic acid or arginine to stop codon mutations (E → * or R → *), consistent with the POLE‐mutant mutational signature (semibold). ^†^The amount of DNA available from the EIN in case Q1‐4 was insufficient for molecular inversion probe sequencing. Details of identified driver mutations are provided in supplementary material, Table S7. (C) H&E‐stained section from colorectal adenoma with the results of Sanger sequencing and allelic discrimination polymerase chain reaction for the wild‐type G allele and mutant T allele.

As somatic POLE mutations have been best characterized in endometrial and colorectal cancers, we first examined whether these mutations were present in precursors of these malignancies. Expert histopathological review of 51 POLE‐mutant endometrial cancers revealed four with a concomitant and spatially discrete area of EIN, the precursor of endometrioid carcinoma (supplementary material, Table S2). Microdissection and targeted sequencing of these lesions by use of a 30‐gene molecular inversion probe capture NGS panel (supplementary material, Table S3), a custom 80‐gene Ion Ampliseq Cancer Hotspot panel (supplementary material, Table S4) and Sanger sequencing revealed that, in all cases, the POLE mutation present in the carcinoma was also detectable in the paired precursor (Figure 1A,B; supplementary material, Table S7). Although some other driver mutations were also shared between the precursors and paired cancers (median of four shared mutations per pair, relative to a median of seven mutations per EIN and median of 10 mutations per carcinoma), the progression from EIN to malignancy was associated with both the loss (median of three mutations lost in carcinomas as compared with paired EINs) and, more frequently, gain (median of six mutations gained in carcinomas as compared with paired EINs) of driver mutations (Figure 1A,B; supplementary material, Table S7). Notably, many of the driver mutations gained were replacements of a glutamic acid or arginine codon with a nonsense codon (E → * or R → *), consistent with the characteristic mutational bias associated with POLE mutation (C:G → A:T transversions, in which the mutated cytosine is in the context TCT, and C:G → T:A transitions, in which the mutated cytosine is in the context TCG) 4, 5, 6 (Figure 1B; supplementary material, Table S7).

We were unable to perform a corresponding analysis of colorectal tumours, because a residual precursor is uncommon in colorectal carcinomas. However, screening of 389 colorectal adenomas from 261 patients revealed three (0.8% adenomas; 1.1% patients) with somatic *POLE* mutations (Figure 1C), a frequency concordant with that found in colorectal cancers 11. Unfortunately, the limited amount of DNA available from these lesions precluded analysis of other driver mutations.

### Mutational landscape and driver gene alterations suggest that somatic POLE mutation is an early event in sporadic endometrial and colorectal cancers

**Figure 2:**
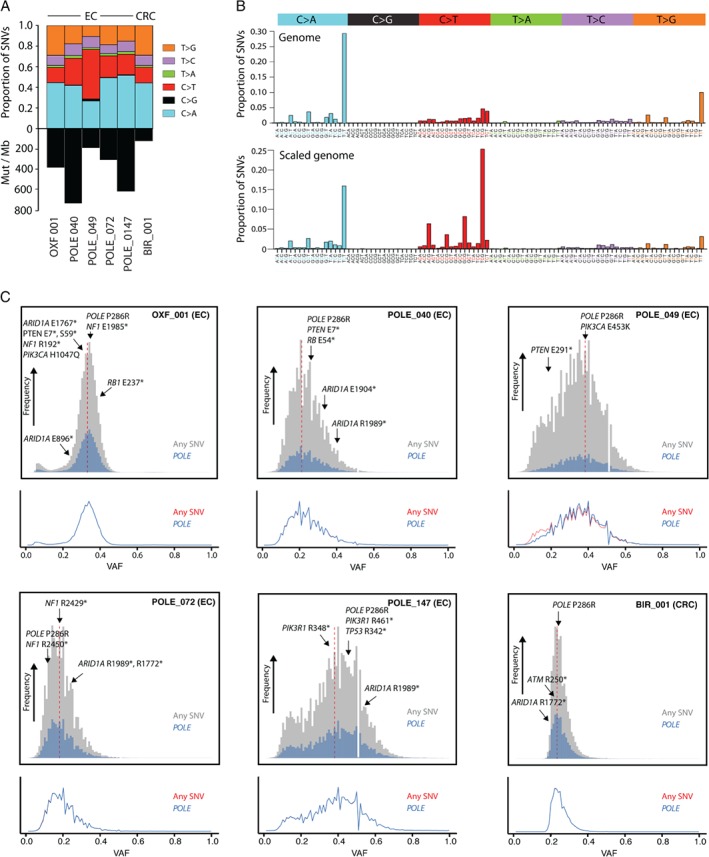
WGS of cancers with POLE mutations. (A) Mutation burden and SNV type determined by WGS of five endometrial cancers (ECs) (Oxf001, POLE_040, POLE_049, POLE_072, and POLE_147) and one colorectal cancer (CRC) (Bir001) with somatic POLE ^P286R^ mutations. (B) Relative proportions of SNV mutations according to trinucleotide context averaged across the six POLE‐mutant cases. The upper panel shows the unscaled proportions across the whole genome, and the lower panel shows the inferred mutational signature in a hypothetical genome for which all trinucleotide frequencies are represented in equal proportions. High‐resolution versions are provided in supplementary material, Figure S1. (C) Frequency histograms and kernel density plots showing the variant allele fraction (VAF) of all SNV mutations, and SNVs that are probably due to POLE mutation (POLE). POLE mutations and other driver gene mutations are highlighted by arrows (details are provided in supplementary material, Table S8). Only mutations in diploid regions of autosomes, and with a coverage of >20×, are shown. The relatively low proportion of SNVs categorized as being due to POLE mutation reflects the stringency of the classification used (see Materials and methods, ‘Mutational signatures’). Vertical red lines indicate the clonal peaks used to calculate cellularity.

To further investigate the timing of *POLE* mutations and their consequences for tumour development, we performed WGS on six cancers (five endometrial; one colorectal), all of which harboured the most common pathogenic *POLE* exonuclease domain variant – a proline to arginine substitution at codon 286 (*POLE* ^P286R^) (Figure 2A). Each showed a substantially elevated mutation burden (122–731 mutations/Mb), and characteristic preponderance of C:G → A:T substitutions in the context TCT (Figure 2A,B; supplementary material, Table S8 and Figure S1) 6. In keeping with their early occurrence, both the *POLE* mutations themselves, and other mutations consistent with the known *POLE* mutational signature (see Materials and methods, ‘Mutational signatures’), appeared to be clonal in all six cases (Figure 2C). This was also the case in 17 of 17 endometrial cancers and 12 of 13 colorectal cancers with pathogenic *POLE* mutations from the TCGA series (supplementary material, Figures S2 and S3). This analysis showed that *POLE* mutations were unlikely to occur as late events after the most recent common ancestor in cancer evolution.

**Figure 3:**
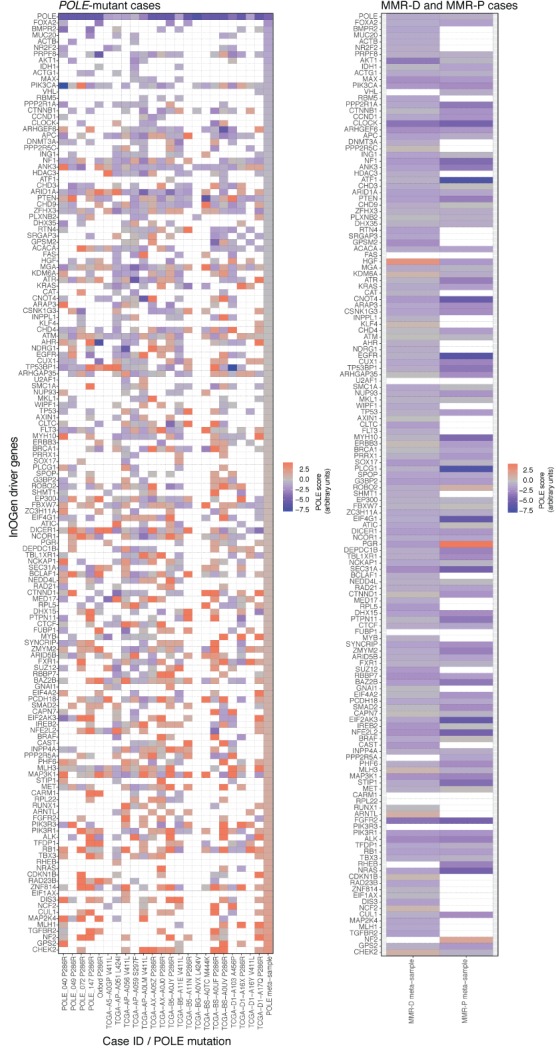
POLE signature mutations in endometrial cancer driver genes. Heatmaps show the modelled probability that mutations in endometrial cancer driver genes (defined on the basis of IntOGen – see Materials and methods, ‘Definition of driver genes’; supplementary material, Table S5) were due to a prior POLE mutation. Results are shown for samples with a pathogenic POLE mutation and MMR‐D and MMR‐P comparators. Each non‐synonymous mutation in a driver gene was assigned a probability that it was caused by the mutational process that generates the distinct POLE mutational signature, rather than by the mutational processes responsible for the consensus mutational signatures of POLE‐wild‐type MMR‐P and MMR‐D tumours (see Materials and methods, ‘POLE consensus mutational signature scores in driver genes’, for details. For each gene/sample combination, a ‘POLE score’ was then calculated as the minimum value of these ratios, and plotted as a heatmap. Scores are shown for both individual POLE‐mutant tumours and the combined POLE‐mutant subgroup; results for tumours within the POLE‐wild‐type MMR‐P and POLE‐wild‐type MMR‐D subgroups are combined for clarity. Scores for POLE itself are shown for reference. Details of mutations are provided in supplementary material, Tables S8 and S9. A high‐resolution version of this figure is provided as supplementary material, Figure S4.

**Figure 4:**
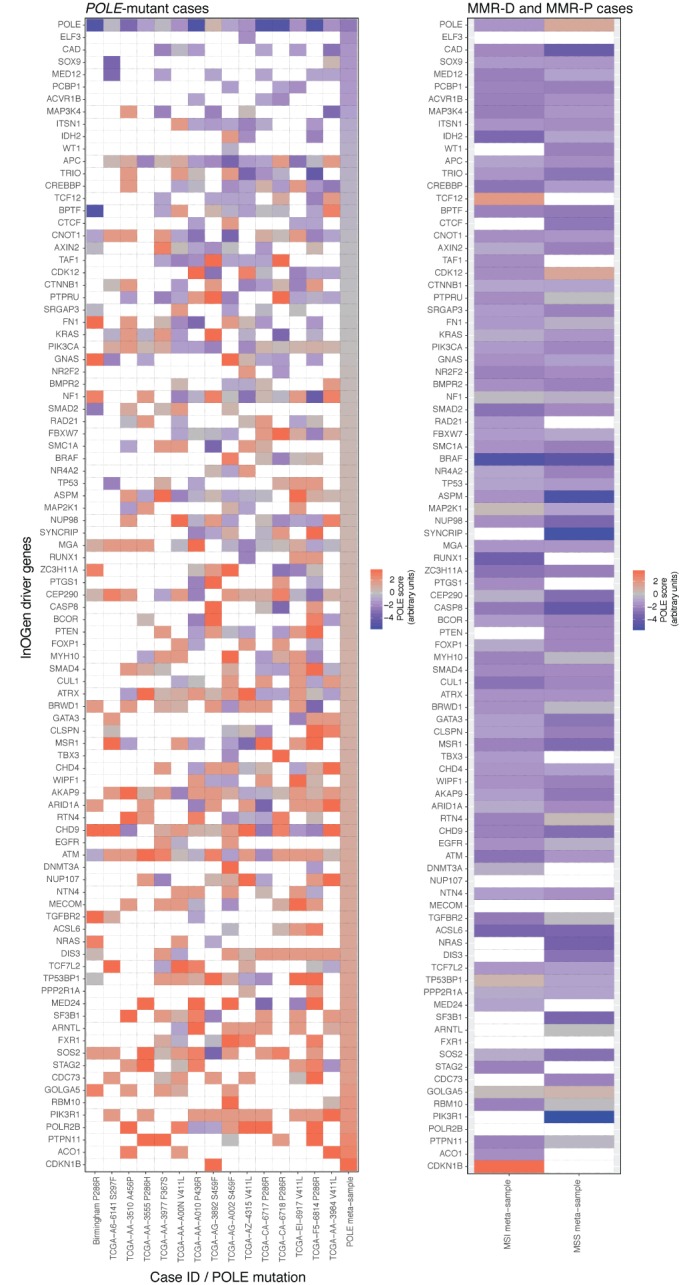
POLE signature mutations in colorectal cancer driver genes. Corresponding heatmaps to those in Figure 3 show the results for known colorectal cancer driver genes (defined on the basis of IntOGen – see Materials and methods, ‘Definition of driver genes’; supplementary material, Table S4). Details of mutations are provided in supplementary material, Tables S8 and S10. A high‐resolution version of this figure is provided as supplementary material, Figure S5.

We next examined the timing of *POLE* mutations in carcinogenesis in more detail by analysis of driver genes, including some that are known to be usually mutated early in the pathogenesis of endometrial or colorectal cancer. To assess the likelihood of mutations in these genes being secondary to an earlier *POLE* mutation, we developed a metric to score them according to the probability that they were caused by the mutational process dominant in *POLE*‐mutant cancers (presumably caused by the *POLE* mutation itself), rather than the mutational processes operative in other tumours (see Materials and methods, ‘*POLE* consensus mutational signature scores in driver genes’ for details). For this analysis, we combined our cohort of *POLE*‐mutant tumours with *POLE*‐mutant cases from TCGA, using MMR‐P and MMR‐D TCGA cases as comparators. Strikingly, in *POLE*‐mutant tumours, almost all known cancer driver genes showed evidence of the *POLE* consensus mutational signature, with the notable exception of *POLE* itself (Figures 3 and 4; supplementary material, Tables S8–S10 and Figures S4 and S5), consistent with the postulate that the *POLE* signature is a direct effect of the polymerase proofreading mutation. In contrast, MMR‐P and MMR‐D tumours rarely showed evidence of the *POLE* consensus mutational signature (Figures 3 and 4; supplementary material, Tables S8–S10). In total, among 206 endometrial and/or colorectal cancer driver genes examined in the cases from the combined endometrial and colorectal cancer cohorts, 50% (1065/2118) of those in *POLE*‐mutant samples had a *POLE* signature score of >0, as compared with 14% (628/4427) in MMR‐D and MMR‐P cancers (*p* < 1 × 10^–26^).

To minimize the possibility of confounding by non‐pathogenic mutations in the complete set of driver genes, we repeated these analyses considering only manually curated, high‐confidence pathogenic mutations, and obtained similar results (*p* < 1 × 10^–26^; supplementary material, Figures S6 and S7). As mutation of the tumour suppressor genes *PTEN* and *APC* are well recognized as early, if not initiating, events in the pathogenesis of endometrial and colorectal cancers, respectively, we specifically examined whether somatic variants in these genes varied according to tumour *POLE* mutation status. Among high‐confidence pathogenic *PTEN* mutations in endometrial cancers, the proportion with *POLE* consensus mutational signature scores of >0 was substantially and significantly greater among *POLE*‐mutant cases than among MMR‐P and MMR‐D tumours [10 of 14 (71.4%) versus 14 of 82 (17.1%) mutations, respectively; *p* = 7.8 × 10^–3^, Fisher's exact test]. Analysis of high‐confidence pathogenic *APC* mutations in colorectal cancers revealed similar results [corresponding proportions nine of 14 (64.3%) versus 10 of 69 (14.5%) mutations; *p* = 0.012, Fisher's exact test].

Further analysis of these cohorts and of targeted sequencing data from an additional series of endometrial cancers from the LUMC, including 32 *POLE*‐mutant tumours, confirmed the over‐representation of E → *, R → * and arginine to glutamine substitutions (R → Q) among *POLE*‐mutant cases, concordant with the results from the paired endometrial lesions and consistent with the known trinucleotide bias of the *POLE* mutational signature (supplementary material, Figures S8–S10 and Tables S7–S11). Interestingly, this was evident not only in well‐characterized driver genes such as *PTEN* in endometrial cancer and *APC* in colorectal cancer, as noted above, but also in recurrent, clonal driver mutations that are rarely found in that tumour type. For example, in the combined TCGA/LUMC endometrial cancer cohorts, truncating mutations in the tumour suppressors *APC*, *NF1* and *RB1* were very rare in *POLE‐*wild‐type tumours (1.1%, 1.5%, and 1.5%, respectively), but common among *POLE*‐mutant cases (38.8%, 34.7%, and 34.7%, respectively; *p* < 0.001 for each comparison, Fisher's exact test), in which they almost invariably occurred at glutamic acid or arginine codons (supplementary material, Figures S8–S10, and Tables S9 and S11).

Collectively, these data suggest that somatic *POLE* mutation occurs early in endometrial and colorectal cancers, and that its attendant mutator phenotype defines a distinct pathway of carcinogenesis from the initial stages of this process.

### Somatic POLE mutations are associated with a prominent T‐cell infiltrate in both precancerous and cancerous lesions

**Figure 5:**
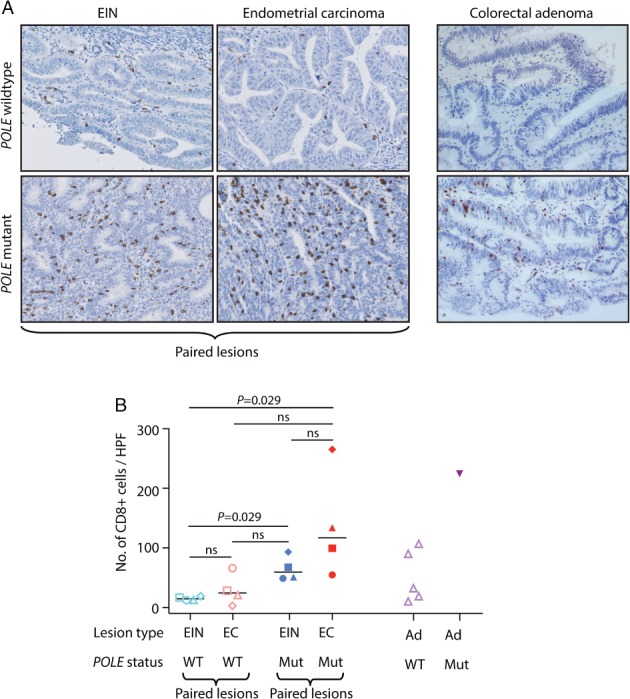
T‐cell infiltrate in POLE‐mutant precursor lesions. (A) Representative immunohistochemical images for the cytotoxic T‐cell marker CD8 in EINs and paired concomitant endometrioid adenocarcinomas and in colorectal adenomas according to POLE mutation status. (B) Quantification of CD8^+^ infiltrate density (number of CD8^+^ cells per HPF calculated as the mean of 10 HPFs) in POLE‐wild‐type and POLE‐mutant paired EIN and endometrial carcinoma (EC) (n = 4 EIN–carcinoma pairs for each genotype) and in POLE‐wild‐type and POLE‐mutant colorectal adenomas (Ad) (n = 5 POLE‐wild‐type lesions, and the single POLE‐mutant adenoma informative for analysis). Symbols (square, circle, triangle and diamond) correspond to paired EIN and endometrial carcinomas for POLE‐wild‐type (open symbols) and POLE‐mutant (closed symbols) cases. For colorectal adenomas, open and closed triangles correspond to unpaired POLE‐wild‐type and POLE‐mutant adenomas respectively. Statistical comparisons in (B) were performed with an unadjusted Mann–Whitney U‐test. Mut, mutant; ns, not significant; WT, wild type.

Somatic *POLE* mutations in endometrial and colorectal cancers are associated with enhanced tumour immunogenicity and a favourable prognosis 11, 14, 15. We speculated that the early acquisition of somatic *POLE* mutations would cause rapid acquisition of mutations, some of which would produce neoantigens capable of eliciting an antitumour immune response. Consistent with this prediction, all *POLE*‐mutant EINs showed a prominent CD8^+^ infiltrate (Figure 5A), which was significantly greater than that in *POLE*‐wild‐type EINs (median 59.4 versus 14.8 CD8^+^ cells per HPF; *p* = 0.029, Mann–Whitney *U*‐test), and exceeded that observed in the *POLE‐*wild‐type endometrial carcinomas, although this difference was not statistically significant (median 59.4 versus 24.7 CD8^+^ cells per HPF; *p* = 0.11) (Figure 5B). The increased CD8^+^ cell density in *POLE*‐mutant EINs could not obviously be explained by other factors such as patient age, or the stage or grade of the paired carcinoma (supplementary material, Table S2). In contrast, the differences in CD8^+^ density between EINs and paired carcinomas among both *POLE*‐wild‐type and *POLE*‐mutant cases were less marked (median 14.8 versus 24.7, *p* = 0.34, and 59.4 versus 116.9, *p* = 0.11, respectively). The single *POLE*‐mutant colorectal adenoma for which IHC was possible also showed a dense CD8^+^ infiltrate (154.9 versus median 34.0 CD8^+^ cells per HPF) (Figure 5A,B).

### Somatic POLE mutations in colorectal cancer are associated with an enhanced predicted clonal neoantigen burden

**Figure 6:**
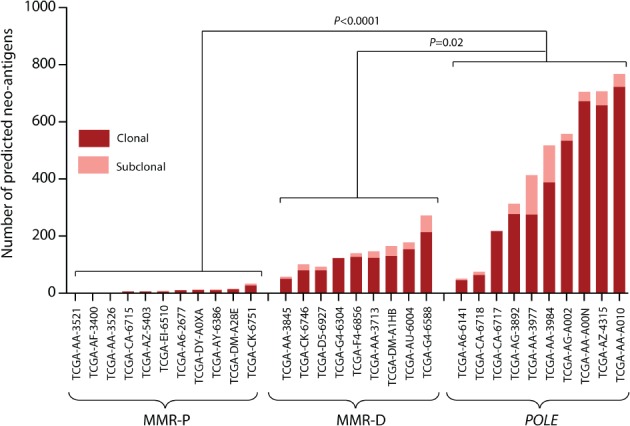
Clonality of predicted neoantigens in POLE‐mutant colorectal cancers. Neoantigens were predicted on the basis of the binding affinity of mutant peptides for patient class I HLA molecules, and were assigned clonal or subclonal status (see Materials and methods, ‘Clonality of POLE mutations’). The numbers of clonal and subclonal neoantigens for POLE‐wild‐type MMR‐P, POLE‐wild‐type MMR‐D and POLE‐mutant colorectal cancers from the TCGA series are shown. Cases in each molecular subgroup were selected to provide broadly similar proportions of disease stages and patient ages: molecular subgroups did not differ significantly in either parameter. Comparison of the clonal neoantigen burden between groups was performed with an unadjusted Mann–Whitney U‐test.

Recent data have shown that the presence of predicted neoantigens within the major tumour clone correlates with the benefit of immune checkpoint inhibitor therapy 18. As the limited amount of FFPE‐derived DNA from precursor lesions was inadequate for clonality analysis and neoantigen prediction, we examined predicted neoantigen clonality in a subset of TCGA colorectal cancers including MMR‐P, MMR‐D and *POLE*‐mutant subtypes, broadly matched for patient age and tumour stage. We used an approach similar to that in our previous reports 11, 14, modified to incorporate patient‐specific HLA haplotypes obtained with OptiType 35 and estimates of tumour clonality derived from analysis of variant allele frequencies (see Materials and methods, ‘Clonal neoantigen prediction’). Analysis of our combined cohort with this pipeline confirmed that *POLE*‐mutant colorectal cancers harboured a substantially greater number and density of predicted clonal neoantigens (0.12/Mb) than tumours lacking *POLE* mutations, including both MMR‐P cases (0.0029/Mb; *p* = 0.0002, Mann–Whitney *U*‐test) and hypermutated MMR‐D cases (0.044/Mb; *p* = 0.03) (Figure 6; supplementary material, Figure S11).

## Discussion

In this article, we have presented multiple lines of evidence to show that pathogenic, somatic *POLE* mutations are usually early and, as far as we can detect, initiating events in endometrial and colorectal tumourigenesis. We show that the acquisition of *POLE* mutation causes a distinct pattern of mutations in cancer driver genes, a substantially increased mutation burden, and an enhanced immune response, detectable even in precancerous lesions. Furthermore, we show that early somatic *POLE* mutations are likely to cause an enrichment of clonal neoantigens that may explain the good prognosis of cancers carrrying these variants, and their excellent response to immune checkpoint inhibitors.

*APC* mutation has traditionally been regarded as the initiating event in sporadic colorectal cancers that develop along the canonical pathway 19, and *PTEN* mutation is thought to play a similar role in sporadic endometrioid endometrial cancers 36. Our evidence suggests that, in sporadic colorectal and endometrial cancers with pathogenic somatic *POLE* mutations, the *POLE* mutation is antecedent to either of these events. The consequent mutator phenotype that it causes influences the types of mutation in these genes and those of the other earliest driver mutations in these cancers, as well as determining their overall mutational landscape 6. Whether any of these *POLE*‐induced driver mutations represent targetable alterations will be an important topic for future research. Similarly, although the increased burden of predicted clonal neoantigens in *POLE*‐mutant tumours may explain their enhanced immunogenicity, further work is required to understand the molecular factors that determine this and its therapeutic implications. A further intriguing possibility is that the mutator phenotype and mutational bias drive cancers into an evolutionary cul‐de‐sac of suboptimal fitness. The presence of *APC* mutations as an alternative to *CTNNB1* mutations in some *POLE‐*mutant endometrial cancers is an exemplar, and there are likely to be others, such as *NF1* and *RB1* mutations in endometrial cancer and atypical (Q61P, K117 N, and A146T) *KRAS* mutations in colorectal cancer. Examination of this hypothesis by comparing the oncogenic effects of these uncommon mutations with those of more typical variants in model systems would be of considerable interest.

Our data add to the expanding body of evidence suggesting that the effects of genomic instability in cancer depend upon both its severity and its timing. For example, upregulation of APOBEC cytosine deaminase enzymes is common in many types of cancer, resulting in an increased mutation rate and characteristic mutation spectrum 6. However, APOBEC overexpression often occurs as a late event in advanced tumours, and causes a more modest mutator phenotype than *POLE* mutations 2, 6. Speculatively, these features may explain why the impact of APOBEC on prognosis appears to be more variable than that of *POLE* mutation 37, 38. The early acquisition of somatic *POLE* mutations in sporadic cancers may also help to explain their association with young age at diagnosis, given the prediction that the early gain of a mutator phenotype will accelerate the process of malignant transformation 39.

Our study has limitations. The number of precursor lesions informative for detailed analysis was limited, in keeping with the relative rarity of *POLE* mutations in endometrial cancer, and the frequency with which precancerous and cancerous lesions occur in the same tumour section. Moreover, although the spatial separation of the precancerous and cancerous compartments, and the discordance in molecular alterations between the two components in each case, suggest otherwise, we cannot exclude the possibility that the apparent precursor lesion is, in fact, adenocarcinoma colonizing endometrial glands. It will therefore be important to validate our results in additional cohorts, although we note that a very recent study has documented a pathogenic *POLE* mutation in an endometrial cancer precursor 25. Furthermore, all of our results are based on the analysis of a single sample of each cancer, meaning that the effects of intratumour heterogeneity on the pattern of driver mutations and clonal neoantigens in *POLE*‐mutant tumours require further definition. However, the absence of multiregion sequencing is unlikely to have confounded the principal conclusions of our study regarding the timing of these pathogenic mutations in cancers.

In summary, we show that pathogenic, somatic *POLE* mutations are early, quite possibly initiating, events in sporadic cancers, and strongly shape subsequent tumour evolution. Our observation provides further insights into the distinct biology of these tumours, and may help to explain their increased immunogenicity and excellent prognosis.

## Author contributions statement

DT, TG, DNC, and IT designed the study. DT, IVG, ER, SM, MB, LC, CP, JD, AB, CS, JM, VC, MR, AA, FA, DL, VS, and TB collected data. DT, IVG, ER, MG, LC, CP, AMB, MW, MR, JT, AS, VS, TB, TG, DNC, and IT analysed data. DT, TG, DNC, and IT interpreted data. DNC and IT wrote the manuscript.

**SUPPLEMENTARY MATERIAL ONLINE**

**Supplementary materials and methods**

**Supplementary figure legends**

**Figure S1.** Relative proportion of SNV mutations according to trinucleotide context in six *POLE*‐mutant tumour genomes (high resolution image)

**Figure S2.** Clonality of *POLE* mutations and mutational processes in TCGA endometrial cancers

**Figure S3.** Clonality of *POLE* mutations and mutational processes in TCGA colorectal cancers

**Figure S4.** *POLE* signature mutations in endometrial cancer driver genes (high resolution image)

**Figure S5.** *POLE* signature mutations in colorectal cancer driver genes (high resolution image)

**Figure S6.** *POLE* signature in high‐confidence endometrial cancer driver mutations

**Figure S7.** *POLE* signature in high‐confidence colorectal cancer driver mutations

**Figure S8.** Driver mutations in TCGA endometrial cancers

**Figure S9.** Driver mutations in TCGA colorectal cancers

**Figure S10.** Driver mutations in LUMC endometrial cancers

**Figure S11.** Clonality of neoantigens in TCGA colorectal cancers

**Table S1.** Cohorts analysed and molecular analyses performed

**Table S2.** Details of cases used for molecular analyses

**Table S3.** Genes included in custom molecular inversion probe panel

**Table S4.** Genes included in custom Ion AmpliSeq Cancer Hotspot Panel

**Table S5.** List of IntOGen endometrial cancer driver genes used in this study

**Table S6.** List of IntOGen colorectal cancer driver genes used in this study

**Table S7.** Driver mutations detected in paired endometrial intraepithelial neoplasias (EIN) and endometrial carcinomas

**Table S8.** Driver mutations in *POLE*‐mutant cancers analysed by whole genome sequencing

**Table S9.** Driver mutations in TCGA endometrial cancers by tumour molecular subgroup

**Table S10.** Driver mutations in TCGA colorectal cancers by tumour molecular subgroup

**Table S11.** Driver mutations in endometrial cancers analysed by Ion Ampliseq Comprehensive Cancer Panel

## Supporting information


**Appendix S1.** Supplementary Materials and methods

**Figure S1. Relative proportion of SNV mutations according to trinucleotide context in six POLE‐mutant tumour genomes (high resolution image).**
This is corresponds to Figure 2B and is provided for clarity of all labels.

**Figure S2. Clonality of POLE mutations and mutational processes in TCGA endometrial cancers**
Frequency histograms and kernel density plots showing variant allele fraction (VAF) of all SNV mutations, and SNVs likely due to POLE exonuclease domain mutation (POLE). Only mutations in diploid regions of autosomes, and with coverage >20x are shown. The relatively low proportion of SNVs categorised as being due to POLE mutation reflects the stringency of the classification used (see Materials and methods, Mutational signatures). VAF of POLE mutations are highlighted. Vertical red line indicates clonal peak used to calculate cellularity.

**Figure S3. Clonality of POLE mutations and mutational processes in TCGA colorectal cancers**
Frequency histograms and kernel density plots showing variant allele fraction (VAF) of all SNV mutations, and SNVs likely due to POLE exonuclease domain mutation (POLE). Only mutations in diploid regions of autosomes, and with coverage >20x are shown. The relatively low proportion of SNVs categorised as being due to POLE mutation reflects the stringency of the classification used (see Materials and methods, Mutational signatures)VAF of POLE mutations are highlighted. Vertical red line indicates clonal peak used to calculate cellularity.

**Figure S4**. POLE signature mutations in endometrial cancer driver genes (high resolution image).
This is corresponds to Figure 3 and is provided for clarity of all labels.

**Figure S5**. POLE signature mutations in colorectal cancer driver genes (high resolution image).
This is corresponds to Figure 4 and is provided for clarity of all labels.

**Figure S6. POLE signature in high‐confidence endometrial cancer driver mutations**
Corresponding heatmap to Figure 3, limited to high‐confidence endometrial cancer driver mutations. High confidence driver mutations were defined as those causing protein truncations known to perturb function of tumour suppressors and missense variants at recurrently‐mutated hotspot codons in either tumour suppressors and oncogenes. Each driver gene mutation was assigned a probability that it was caused by the mutational process that generates the distinct POLE mutational signature, rather than by the mutational processes responsible for the consensus mutational signatures of POLE‐wild‐type DNA mismatch repair proficient (MMR‐P) and mismatch repair deficient (MMR‐D) tumours (see Materials and methods, POLE consensus mutational signature scores in driver genes, for details. For each gene/sample combination, a ‘POLE‐score’ was then calculated as the base two logarithm of the minimum value of these ratios, and plotted as a heatmap. Scores are shown for both individual POLE‐mutant tumours and the combined POLE‐mutant subgroup; results for tumours within the POLE‐wild‐type, mismatch repair proficient (MMR‐P) and POLE‐wild‐type, mismatch repair deficient (MMR‐D) subgroups are combined for clarity.

**Figure S7. POLE signature in high‐confidence colorectal cancer driver mutations**
Corresponding heatmap to Figure 4, limited to high‐confidence endometrial cancer driver mutations. High confidence driver mutations were defined as those causing protein truncations known to perturb function of tumour suppressors and missense variants at recurrently‐mutated hotspot codons in either tumour suppressors and oncogenes. Each driver gene mutation was assigned a probability that it was caused by the mutational process that generates the distinct POLE mutational signature, rather than by the mutational processes responsible for the consensus mutational signatures of POLE‐wild‐type DNA mismatch repair proficient (MMR‐P) and mismatch repair deficient (MMR‐D) tumours (see Materials and methods, POLE consensus mutational signature scores in driver genes, for details. For each gene/sample combination, a ‘POLE‐score’ was then calculated as the base two logarithm of the minimum value of these ratios, and plotted as a heatmap. Scores are shown for both individual POLE‐mutant tumours and the combined POLE‐mutant subgroup; results for tumours within the POLE‐wild‐type, mismatch repair proficient (MMR‐P) and POLE‐wild‐type, mismatch repair deficient (MMR‐D) subgroups are combined for clarity.

**Figure S8. Driver mutations in TCGA endometrial cancers**
Comparison of mutation type and frequency in selected driver genes according to tumour molecular subtype. POLE wt – POLE‐wild‐type group includes tumour irrespective of DNA mismatch repair status. POLE mut – pathogenic somatic POLE exonuclease domain mutations. Glutamic acid to stop mutations (E→*) occur when a glutamic acid codon (GAG or GAA) is preceded by an A (e.g. AGAG or AGAA), creating an AGA trinucleotide which is commonly mutated to ATA in POLE‐mutant tumours, causing a stop codon (TAG or TAA). Arginine to stop mutations (R→*) occur when the POLE hotspot trinucleotide TCG is followed by an A, resulting in a TCGA to TTGA mutation. Arginine to glutamine substitutions (R→Q) occur when the reverse complement of this hotspot, CGA is mutated to CAA.

**Figure S9. Driver mutations in TCGA colorectal cancers**
Comparison of mutation type and frequency in selected driver genes according to tumour molecular subtype. POLE wt – POLE‐wild‐type group includes tumour irrespective of DNA mismatch repair status. POLE mut – pathogenic somatic POLE exonuclease domain mutations. Glutamic acid to stop mutations (E→*) occur when a glutamic acid codon (GAG or GAA) is preceded by an A (e.g. AGAG or AGAA), creating an AGA trinucleotide which is commonly mutated to ATA in POLE‐mutant tumours, causing a stop codon (TAG or TAA). Arginine to stop mutations (R→*) occur when the POLE hotspot trinucleotide TCG is followed by an A, resulting in a TCGA to TTGA mutation. Arginine to glutamine substitutions (R→Q) occur when the reverse complement of this hotspot, CGA is mutated to CAA.

**Figure S10. Driver mutations in LUMC endometrial cancers**
Comparison of mutation type and frequency in selected driver genes according to tumour molecular subtype in a cohort of FFPE tumours from the Leiden University Medical Centre (LUMC). POLE wt – POLE‐wild‐type group includes tumour irrespective of DNA mismatch repair status. POLE mut – pathogenic somatic POLE exonuclease domain mutations. Glutamic acid to stop mutations (E→*) occur when a glutamic acid codon (GAG or GAA) is preceded by an A (e.g. AGAG or AGAA), creating an AGA trinucleotide which is commonly mutated to ATA in POLE‐mutant tumours, causing a stop codon (TAG or TAA). Arginine to stop mutations (R→*) occur when the POLE hotspot trinucleotide TCG is followed by an A, resulting in a TCGA to TTGA mutation. Arginine to glutamine substitutions (R→Q) occur when the reverse complement of this hotspot, CGA is mutated to CAA.

**Figure S11. Clonality of neoantigens in TCGA colorectal cancers**
Frequency histograms for cases corresponding to Figure 6 showing variant allele fraction (VAF) of all SNV mutations and predicted neo‐antigens. Only mutations in diploid regions of autosomes, and with coverage >20x were considered. Vertical red line indicates inferred clonal peak used to calculate cellularity.

**Table S1**. Cohorts analysed and molecular analyses performed

**Table S2**. Details of cases used for molecular analyses

**Table S3**. Genes included in custom molecular inversion probe panel

**Table S4**. Genes included in custom Ion AmpliSeq Cancer Hotspot Panel

**Table S5**. List of IntOGen endometrial cancer driver genes used in this study

**Table S6**. List of IntOGen colorectal cancer driver genes used in this study

**Table S7**. Driver mutations detected in paired endometrial intraepithelial neoplasias (EIN) and endometrial carcinomas

**Table S8**. Driver mutations in POLE‐mutant cancers analysed by whole genome sequencing

**Table S9**. Driver mutations in TCGA endometrial cancers by tumour molecular subgroup

**Table S10**. Driver mutations in TCGA colorectal cancers by tumour molecular subgroup

**Table S11**. Driver mutations in endometrial cancers analysed by Ion Ampliseq Comprehensive Cancer Panel
