# Supplementary material for: Somatic POLE exonuclease domain mutations are early events in sporadic endometrial and colorectal carcinogenesis, determining driver mutational landscape, clonal neoantigen burden and immune response
Source: J Pathol. 2018 Apr 30;245(3):283–96. doi: 10.1002/path.5081 (PMC6032922; doi:10.1002/path.5081)
Supplement: Supplementary file 1 — Appendix S1. Supplementary Materials and methods [file PATH-245-283-s012.docx]

**Supplementary Materials and methods**

Reference numbers refer to the main text list

*DNA sequencing*

Endometrial epithelial neoplasias (EIN) and paired endometrial cancers were analysed by a custom molecular inversion probe (MIP) panel covering 30 genes commonly mutated in endometrial and colorectal cancers (supplementary material, Table S3), and a clinical-grade ThermoFisher Ion AmpliSeq Cancer Hotspot Panel v2, which includes 376 amplicons across 80 genes (supplementary materials, Table S4). For the MIP panels, libraries were sequenced on an Illumina NextSeq and aligned to GrCh37 using BWA mem. Variants were called using LoFreq [28] and annotated by VEP [33]. Only variants with VAF >0.1 were used for analysis. For the Ion AmpliSeq panel, library preparation was performed according to the manufacturer’s protocol using 50 ng of genomic DNA. Libraries were normalized to 60 pM, loaded on a PI chip using the Ion Chef System (ThermoFisher), and sequenced with the Ion Proton System (ThermoFisher). The generated reads were aligned to the human reference genome (GRCh37) using the TMAP 5.0.7 software with default parameters (https://github.com/iontorrent/TS). The Ion Torrent specific variant caller was used for variant calling (ThermoFisher) and Geneticist Assistant (Softgenetics) for variant interpretation. Variants were removed with a variant allele frequency of <0.1 and/or with a coverage of <100. Called mutations were visually inspected using the Integrative Genomics Viewer (IGV) software (<http://software.broadinstitute.org/software/igv/>). All *POLE* mutations were confirmed by Sanger sequencing as reported previously [30], with the exception of one EIN with low DNA yield, for which competitive allele-specific PCR (LGC Genomics) assay was used (details of primers and reaction conditions available on request). FFPE colorectal adenomas were screened for somatic *POLE* exonuclease domain mutations by a combination of Sanger sequencing and competitive allele-specific PCR as described previously 11].

DNA from fresh frozen endometrial tumours and paired normal samples for whole genome sequencing (WGS) was quantified using the Qubit® 2.0 fluorometer (Life Technologies, Paisley, UK), and fragmented using the Covaris M220 ultrasonicator (Covaris, Inc., Woburn, MA, UK) to an average fragment size of 250–300 bp. Approximately 50 ng was used as input for the NEBNext® Ultra™ DNA Library Prep Kit for Illumina® (New England Biolabs, Hitchin, UK). Libraries were prepared as per the manufacturer’s guidelines, with size selection for a 250 bp insert size, dual indexing and 9 cycles of library amplification. Libraries were sequenced to a median depth of ~50x on Illumina’s HiSeq X Ten (150 bp paired end reads) at BGI Tech Solutions Ltd, Hong Kong, PR China. Somatic mutations were called from DNA sequencing data using Mutect2 [29]. Variants flagged as ‘PASS’ or ‘clustered_events’ were accepted as somatic. Variants were annotated using Annovar [32]. Copy number profiles were derived using Sequenza [31] for a subset of samples, and manually curated to remove probable model artefacts. DNA from the colorectal cancer was prepared for WGS using the Truseq PCR-free library preparation kit (Illumina) following the manufacturers guidelines and sequenced on an Illumina HiSeq 2500. Sequenced reads were aligned to the GrCh37 reference genome using the Isaac aligner [27], and variant calling performed using Strelka [30].

FFPE endometrial cancers from the LUMC series were analysed using the Lifetech/ThermoFisher Ion AmpliSeq Comprehensive Cancer Panel comprising 409 cancer genes (<http://www.lifetechnologies.com/order/catalog/product/4477685)>. Extracted genomic DNA was quantified by Qubit and library preparation performed according to the manufacturer’s protocol (LifeTechnologies, cat. no. 4477685 and 4480442). Details of PCR conditions and library purification and quantification were as previously reported [40]. Diluted libraries were loaded onto the Ion PI chip v2 BC (cat. no. 4484270) and sequenced on the Ion Proton sequencer (cat. no. 4476610). Signal processing and base calling were performed using the Ion Proton Torrent suite (version 4.4). Variant calling was performed using variantCaller and Ion Reporter software (Thermo). Variant call format (VCF) files were annotated using Variant Effect Predictor (VEP) [33] and filtered to remove variants with (i) read depth of <50, quality of depth (QD) of <2 or fewer than 10 supporting reads; (ii) flow evaluator alternate allele observation count of <20; (iii) genotype quality (GQ) of <90; (iv) variants in homopolymer runs of >5; (v) variants ≤3 bases from the end of an amplicon; (vi) minor allele frequency of >1% in ExAc, 1000 Genomes or UK10K; (vii) a variant allele fraction of <0.1. Data were then manually curated to remove likely artefactual variants (e.g. variant absent from COSMIC, and/or present in more cases than a known endometrial cancer gene such as *PIK3CA* or *PTEN*).

Somatic mutations in TCGA cancers were called from BAMs using Mutect2 [29]. Additional cases from the TCGA COADREAD and UCEC data sets were downloaded as Mutect Mutation Annotation Format (MAF) files from the GDC Data Portal. Variant annotation and model curation was performed as for the WGS cases. Copy number data was downloaded for COADREAD data from the same source. ‘Segment Mean’ (S) was transformed using the following formula to find copy number index (I): I = round((2^S)x2). Segments outside chr6.p with copy number index equal to 2 were assumed to be diploid.
