# Supplementary material for: Somatic POLE exonuclease domain mutations are early events in sporadic endometrial and colorectal carcinogenesis, determining driver mutational landscape, clonal neoantigen burden and immune response
Source: J Pathol. 2018 Apr 30;245(3):283–96. doi: 10.1002/path.5081 (PMC6032922; doi:10.1002/path.5081)

Figure S1. Relative proportion of SNV mutations according to trinucleotide context in six *POLE*-mutant tumour genomes

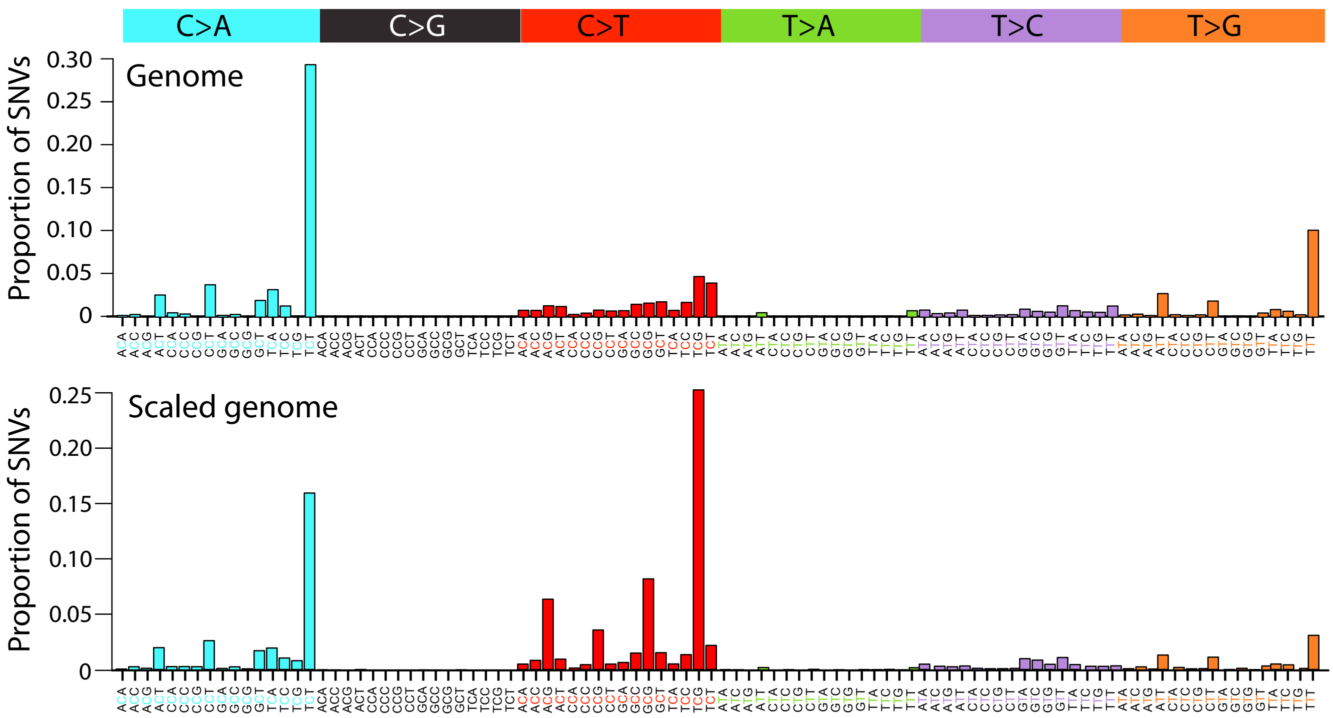

Supplement: Supplementary file 2 — Figure S1. Relative proportion of SNV mutations according to trinucleotide context in six POLE‐mutant tumour genomes (high resolution image). This is corresponds to Figure 2B and is provided for clarity of all labels. [file PATH-245-283-s001.pdf]
