# Supplementary figures and images for: Somatic POLE exonuclease domain mutations are early events in sporadic endometrial and colorectal carcinogenesis, determining driver mutational landscape, clonal neoantigen burden and immune response
Source: J Pathol. 2018 Apr 30;245(3):283–96. doi: 10.1002/path.5081 (PMC6032922; doi:10.1002/path.5081)

Figure S2. Clonality of *POLE* mutations and mutational processes in TCGA endometrial cancers

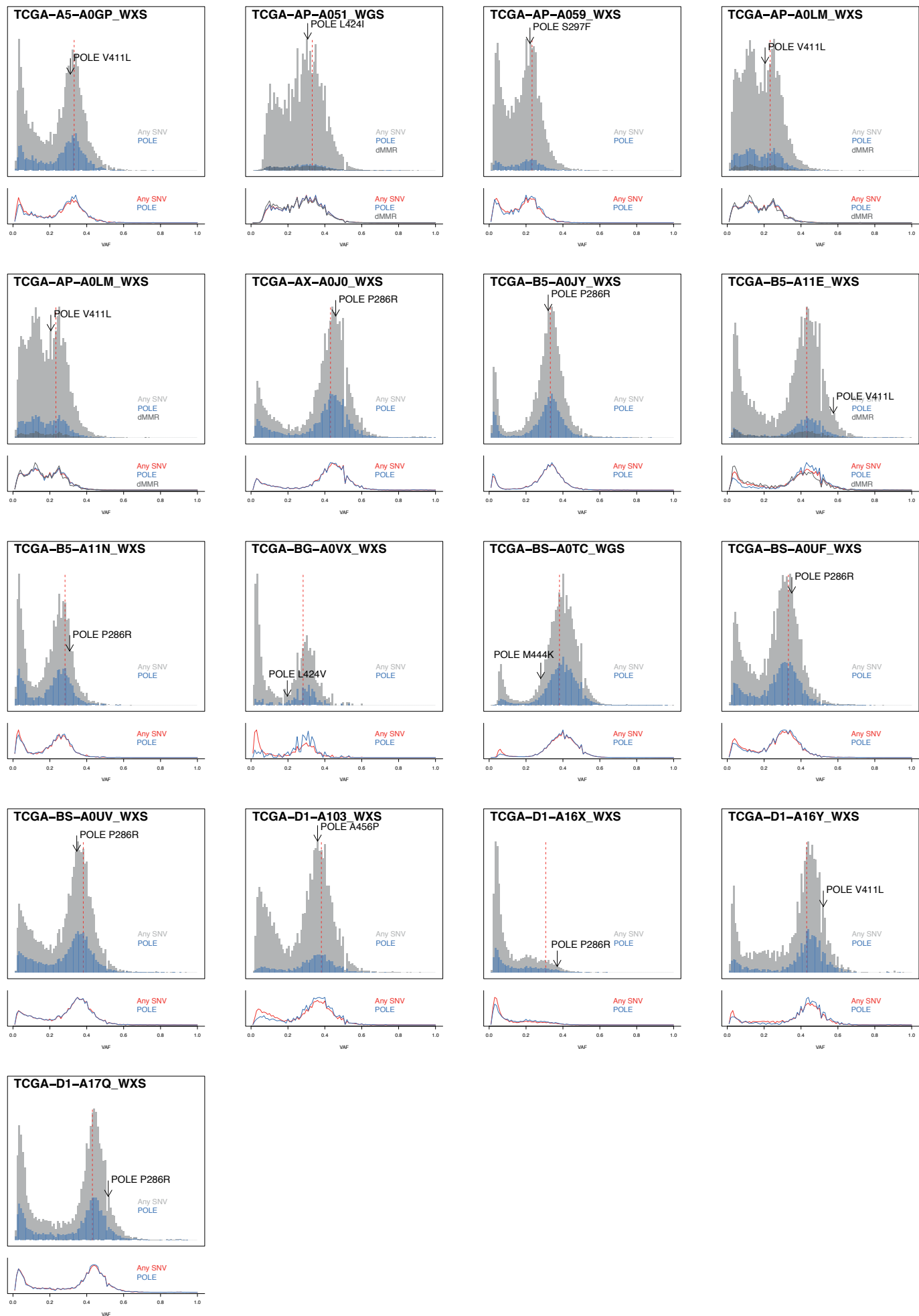

Supplement: Supplementary file 3 — Figure S2. Clonality of POLE mutations and mutational processes in TCGA endometrial cancers Frequency histograms and kernel density plots showing variant allele fraction (VAF) of all SNV mutations, and SNVs likely due to POLE exonuclease domain mutation (POLE). Only mutations in diploid regions of autosomes, and with coverage >20x are shown. The relatively low proportion of SNVs categorised as being due to POLE mutation reflects the stringency of the classification used (see Materials and methods, Mutational signatures). VAF of POLE mutations are highlighted. Vertical red line indicates clonal peak used to calculate cellularity. [file PATH-245-283-s002.pdf]

Figure S3. Clonality of *POLE* mutations and mutational processes in TCGA colorectal cancers

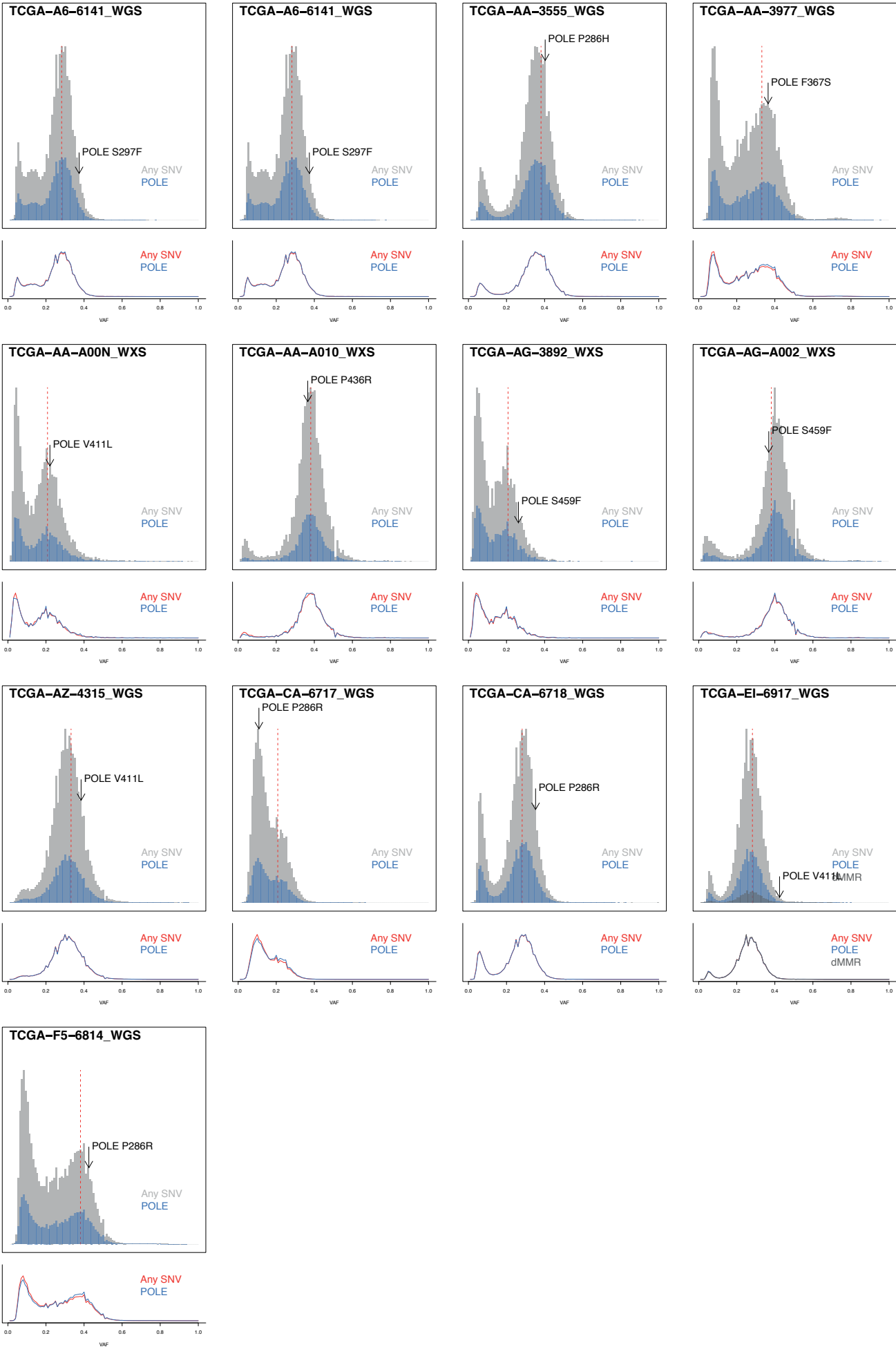

Supplement: Supplementary file 4 — Figure S3. Clonality of POLE mutations and mutational processes in TCGA colorectal cancers Frequency histograms and kernel density plots showing variant allele fraction (VAF) of all SNV mutations, and SNVs likely due to POLE exonuclease domain mutation (POLE). Only mutations in diploid regions of autosomes, and with coverage >20x are shown. The relatively low proportion of SNVs categorised as being due to POLE mutation reflects the stringency of the classification used (see Materials and methods, Mutational signatures)VAF of POLE mutations are highlighted. Vertical red line indicates clonal peak used to calculate cellularity. [file PATH-245-283-s003.pdf]

Figure S4. *POLE* signature mutations in endometrial cancer driver genes

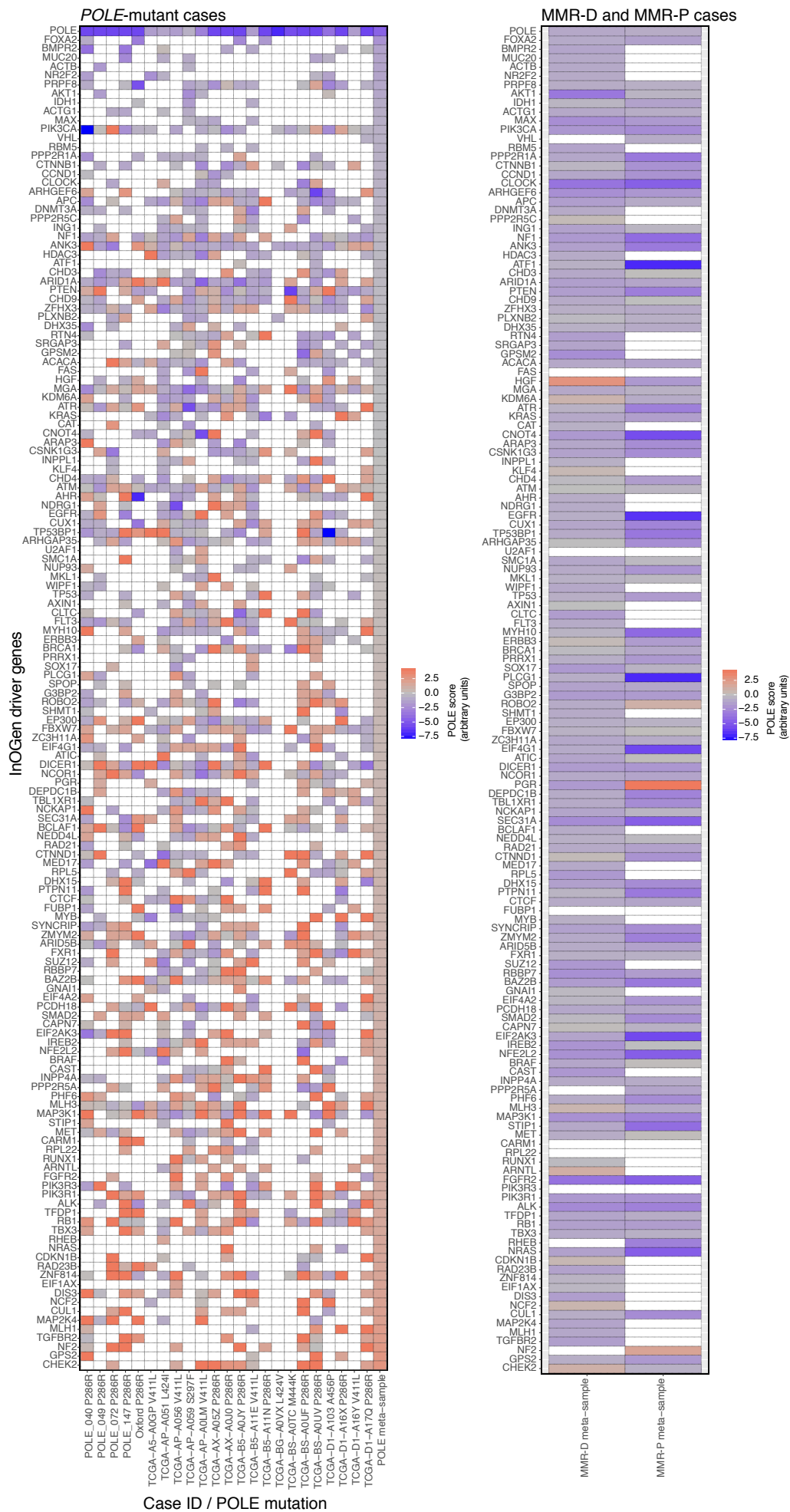

Supplement: Supplementary file 5 — Figure S4. POLE signature mutations in endometrial cancer driver genes (high resolution image). This is corresponds to Figure 3 and is provided for clarity of all labels. [file PATH-245-283-s004.pdf]

Figure S5. *POLE* signature mutations in colorectal cancer driver genes

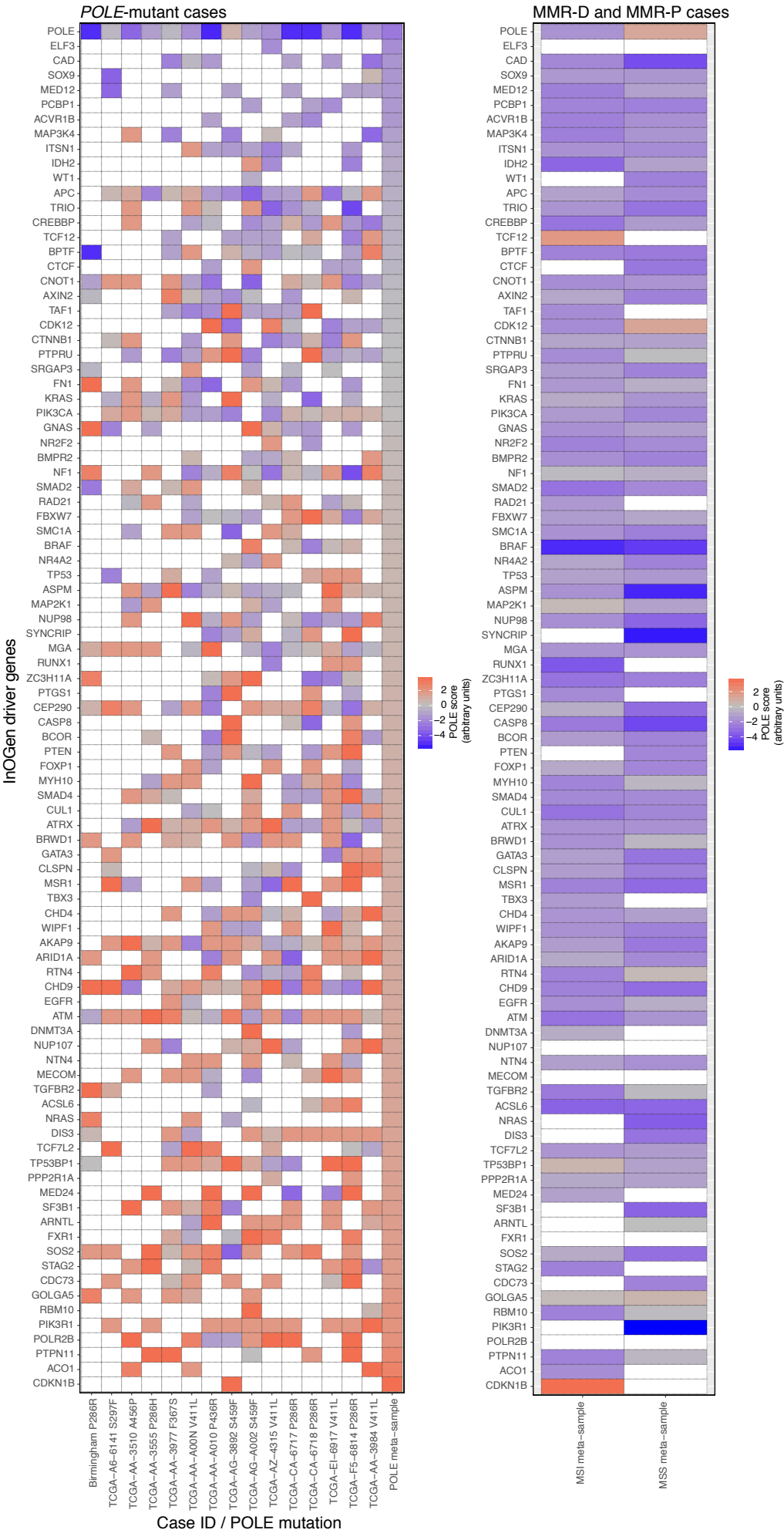

Supplement: Supplementary file 6 — Figure S5. POLE signature mutations in colorectal cancer driver genes (high resolution image). This is corresponds to Figure 4 and is provided for clarity of all labels. [file PATH-245-283-s005.pdf]

Figure S6. *POLE* signature in high-confidence endometrial cancer driver mutations

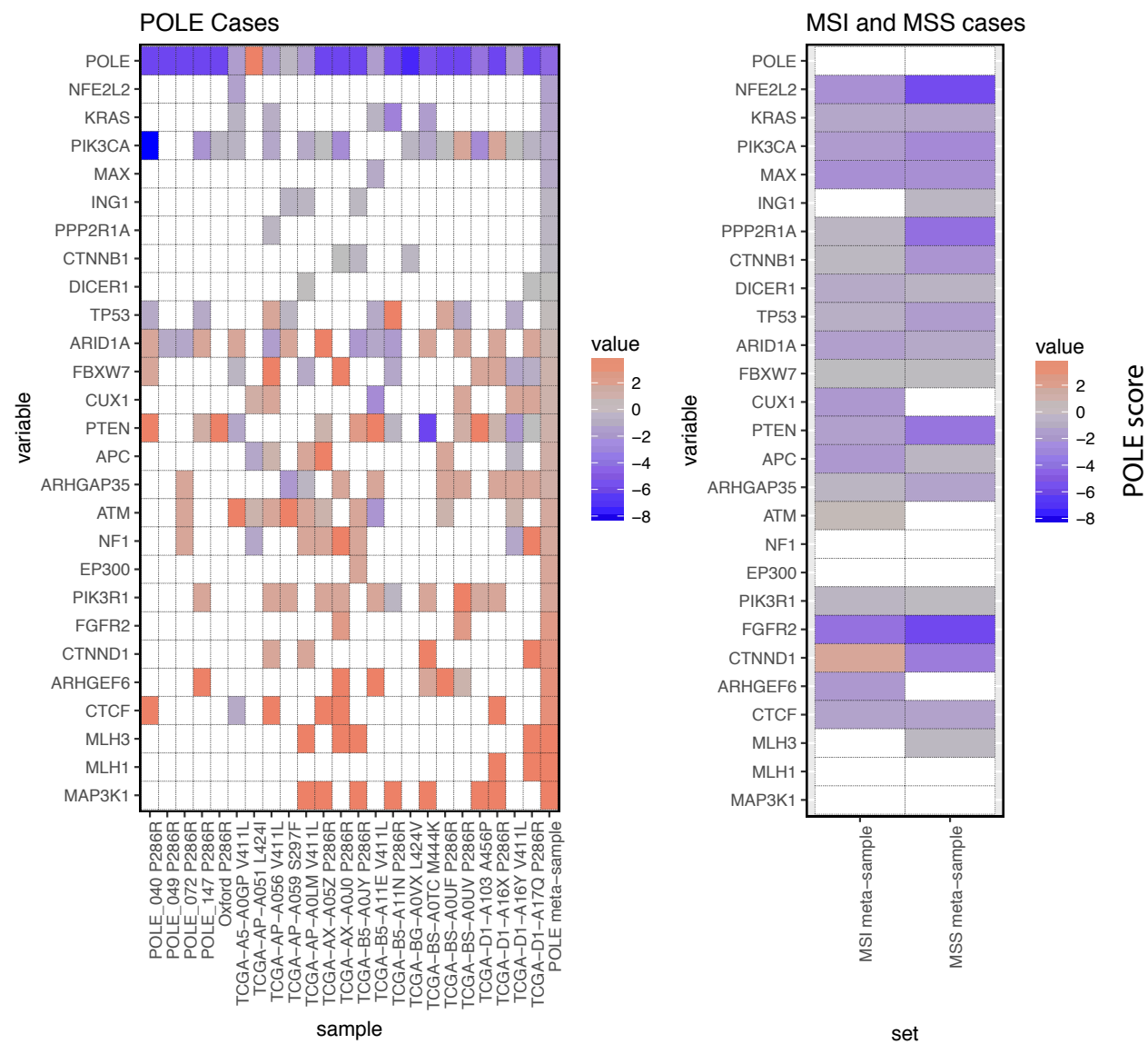

Supplement: Supplementary file 7 — Figure S6. POLE signature in high‐confidence endometrial cancer driver mutations Corresponding heatmap to Figure 3, limited to high‐confidence endometrial cancer driver mutations. High confidence driver mutations were defined as those causing protein truncations known to perturb function of tumour suppressors and missense variants at recurrently‐mutated hotspot codons in either tumour suppressors and oncogenes. Each driver gene mutation was assigned a probability that it was caused by the mutational process that generates the distinct POLE mutational signature, rather than by the mutational processes responsible for the consensus mutational signatures of POLE‐wild‐type DNA mismatch repair proficient (MMR‐P) and mismatch repair deficient (MMR‐D) tumours (see Materials and methods, POLE consensus mutational signature scores in driver genes, for details. For each gene/sample combination, a ‘POLE‐score’ was then calculated as the base two logarithm of the minimum value of these ratios, and plotted as a heatmap. Scores are shown for both individual POLE‐mutant tumours and the combined POLE‐mutant subgroup; results for tumours within the POLE‐wild‐type, mismatch repair proficient (MMR‐P) and POLE‐wild‐type, mismatch repair deficient (MMR‐D) subgroups are combined for clarity. [file PATH-245-283-s006.pdf]

Figure S7. *POLE* signature in high-confidence colorectal cancer driver mutations

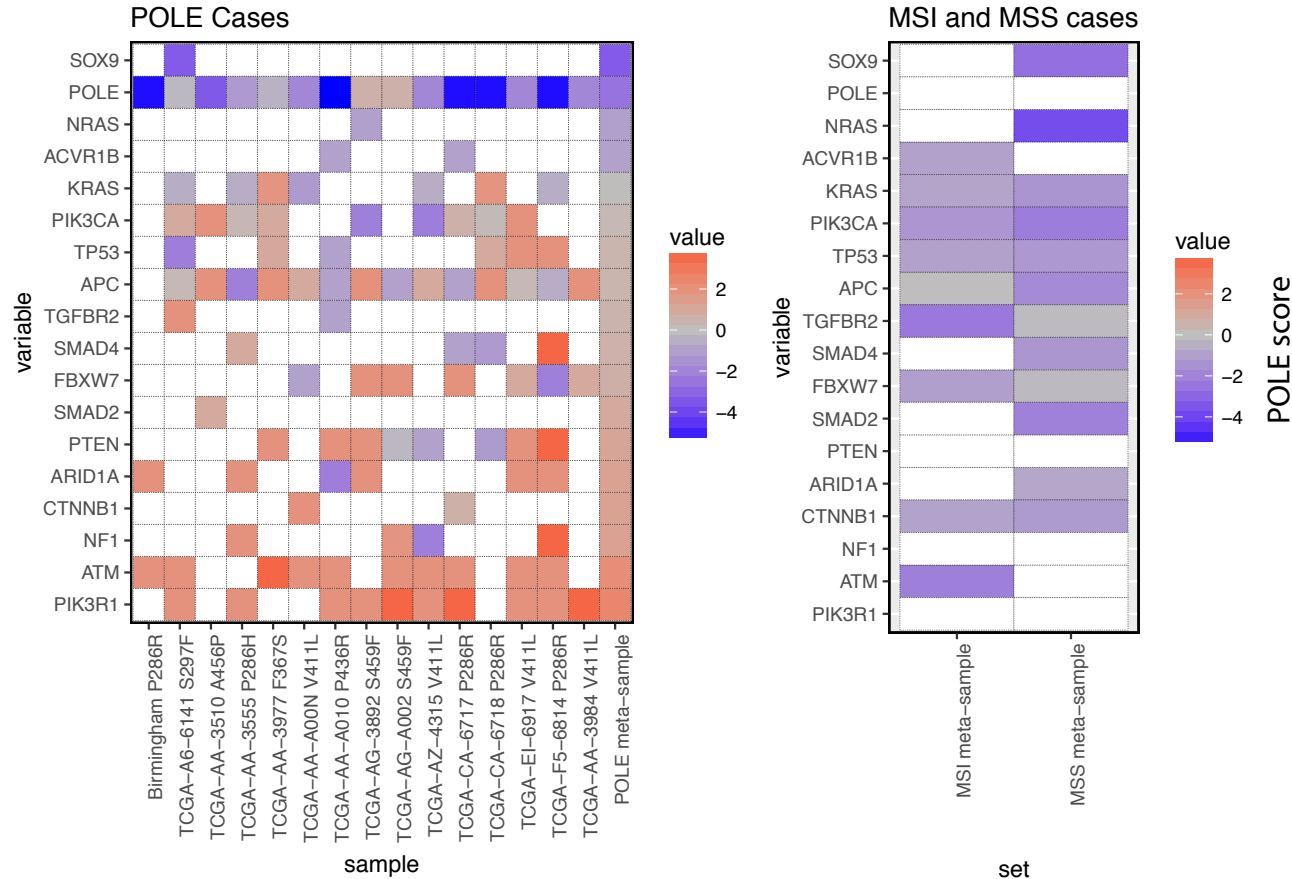

Supplement: Supplementary file 8 — Figure S7. POLE signature in high‐confidence colorectal cancer driver mutations Corresponding heatmap to Figure 4, limited to high‐confidence endometrial cancer driver mutations. High confidence driver mutations were defined as those causing protein truncations known to perturb function of tumour suppressors and missense variants at recurrently‐mutated hotspot codons in either tumour suppressors and oncogenes. Each driver gene mutation was assigned a probability that it was caused by the mutational process that generates the distinct POLE mutational signature, rather than by the mutational processes responsible for the consensus mutational signatures of POLE‐wild‐type DNA mismatch repair proficient (MMR‐P) and mismatch repair deficient (MMR‐D) tumours (see Materials and methods, POLE consensus mutational signature scores in driver genes, for details. For each gene/sample combination, a ‘POLE‐score’ was then calculated as the base two logarithm of the minimum value of these ratios, and plotted as a heatmap. Scores are shown for both individual POLE‐mutant tumours and the combined POLE‐mutant subgroup; results for tumours within the POLE‐wild‐type, mismatch repair proficient (MMR‐P) and POLE‐wild‐type, mismatch repair deficient (MMR‐D) subgroups are combined for clarity. [file PATH-245-283-s007.pdf]

Figure S8. Driver mutations in TCGA endometrial cancers

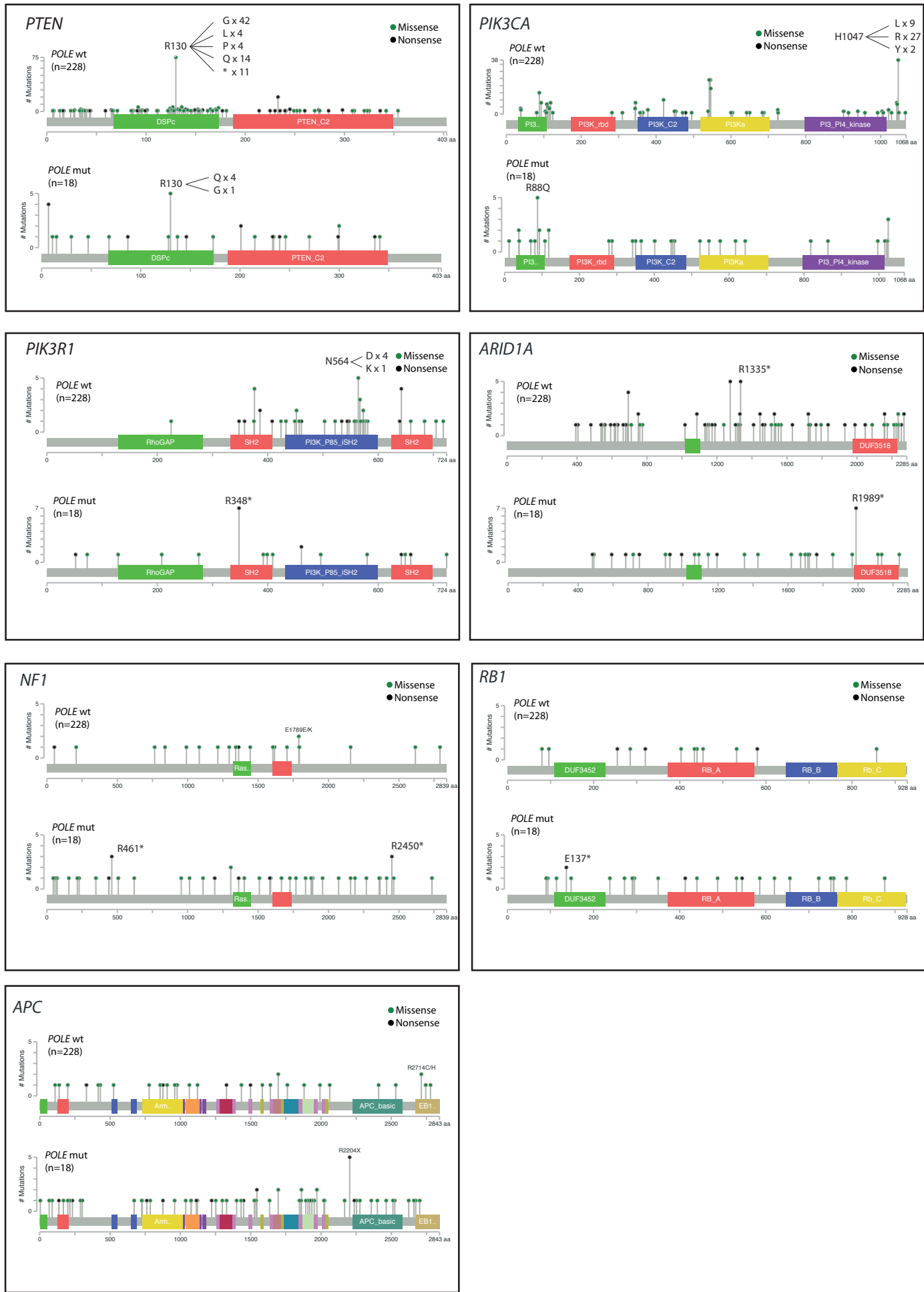

Supplement: Supplementary file 9 — Figure S8. Driver mutations in TCGA endometrial cancers Comparison of mutation type and frequency in selected driver genes according to tumour molecular subtype. POLE wt – POLE‐wild‐type group includes tumour irrespective of DNA mismatch repair status. POLE mut – pathogenic somatic POLE exonuclease domain mutations. Glutamic acid to stop mutations (E→*) occur when a glutamic acid codon (GAG or GAA) is preceded by an A (e.g. AGAG or AGAA), creating an AGA trinucleotide which is commonly mutated to ATA in POLE‐mutant tumours, causing a stop codon (TAG or TAA). Arginine to stop mutations (R→*) occur when the POLE hotspot trinucleotide TCG is followed by an A, resulting in a TCGA to TTGA mutation. Arginine to glutamine substitutions (R→Q) occur when the reverse complement of this hotspot, CGA is mutated to CAA. [file PATH-245-283-s008.pdf]

Figure S9. Driver mutations in TCGA colorectal cancers

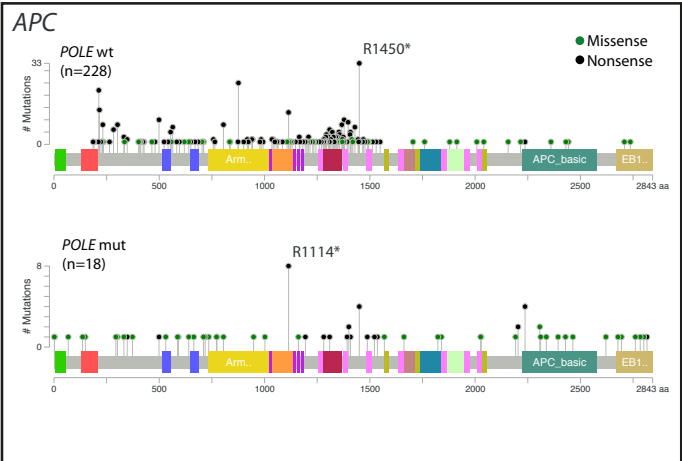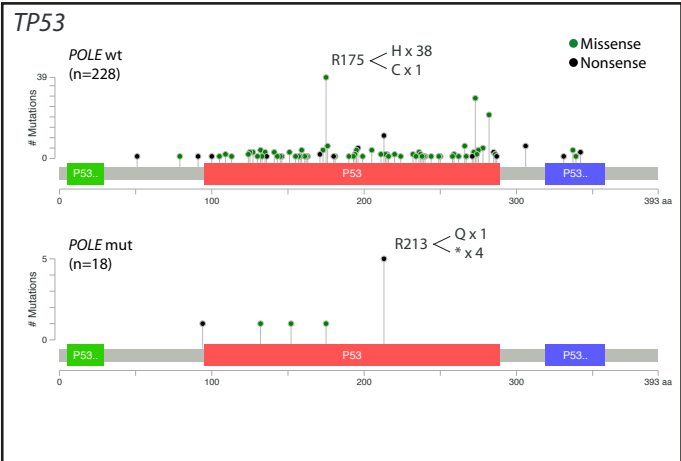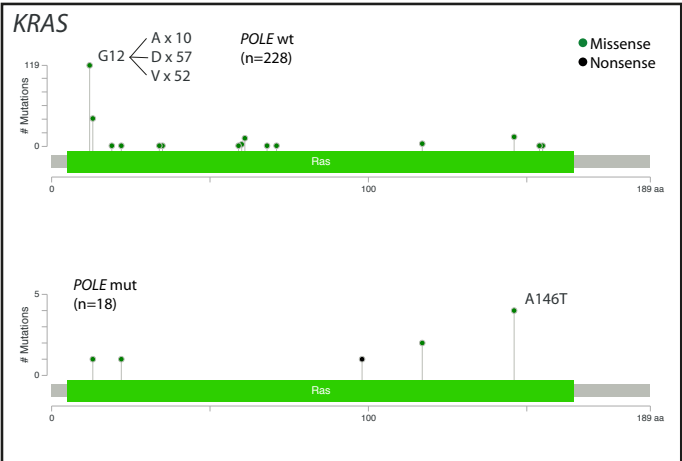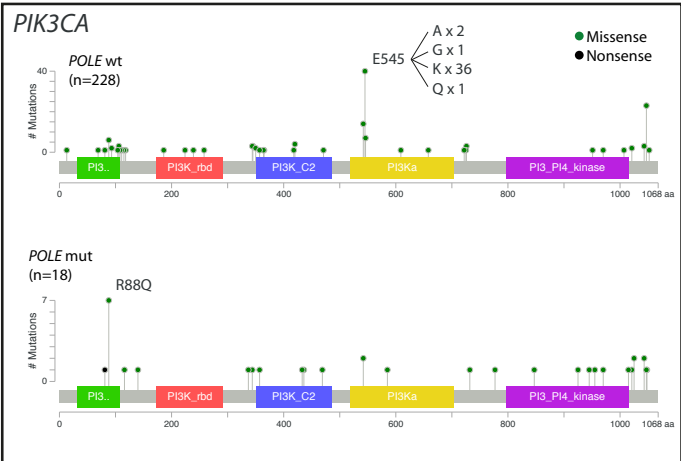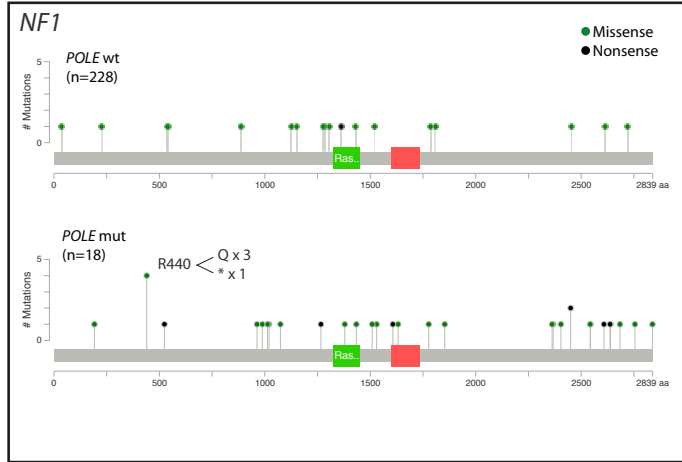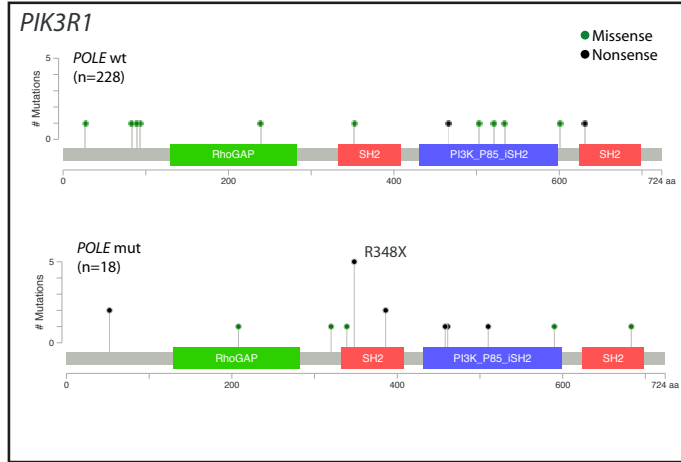

Supplement: Supplementary file 10 — Figure S9. Driver mutations in TCGA colorectal cancers Comparison of mutation type and frequency in selected driver genes according to tumour molecular subtype. POLE wt – POLE‐wild‐type group includes tumour irrespective of DNA mismatch repair status. POLE mut – pathogenic somatic POLE exonuclease domain mutations. Glutamic acid to stop mutations (E→*) occur when a glutamic acid codon (GAG or GAA) is preceded by an A (e.g. AGAG or AGAA), creating an AGA trinucleotide which is commonly mutated to ATA in POLE‐mutant tumours, causing a stop codon (TAG or TAA). Arginine to stop mutations (R→*) occur when the POLE hotspot trinucleotide TCG is followed by an A, resulting in a TCGA to TTGA mutation. Arginine to glutamine substitutions (R→Q) occur when the reverse complement of this hotspot, CGA is mutated to CAA. [file PATH-245-283-s009.pdf]

Figure S10. Driver mutations in LUMC endometrial cancers

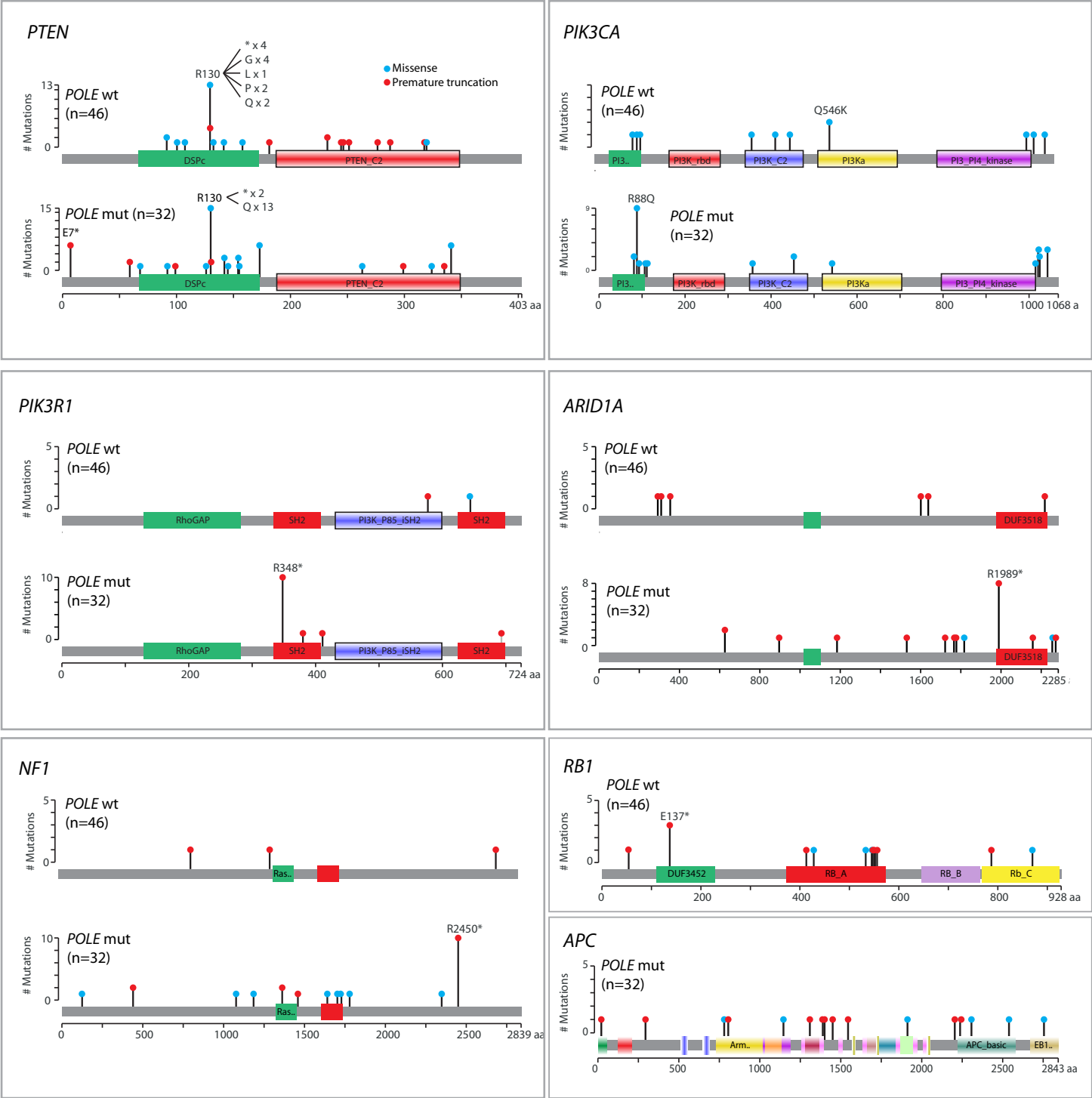

Supplement: Supplementary file 11 — Figure S10. Driver mutations in LUMC endometrial cancers Comparison of mutation type and frequency in selected driver genes according to tumour molecular subtype in a cohort of FFPE tumours from the Leiden University Medical Centre (LUMC). POLE wt – POLE‐wild‐type group includes tumour irrespective of DNA mismatch repair status. POLE mut – pathogenic somatic POLE exonuclease domain mutations. Glutamic acid to stop mutations (E→*) occur when a glutamic acid codon (GAG or GAA) is preceded by an A (e.g. AGAG or AGAA), creating an AGA trinucleotide which is commonly mutated to ATA in POLE‐mutant tumours, causing a stop codon (TAG or TAA). Arginine to stop mutations (R→*) occur when the POLE hotspot trinucleotide TCG is followed by an A, resulting in a TCGA to TTGA mutation. Arginine to glutamine substitutions (R→Q) occur when the reverse complement of this hotspot, CGA is mutated to CAA. [file PATH-245-283-s010.pdf]

Figure S11 Neoantigen clonality in TCGA colorectal cancers

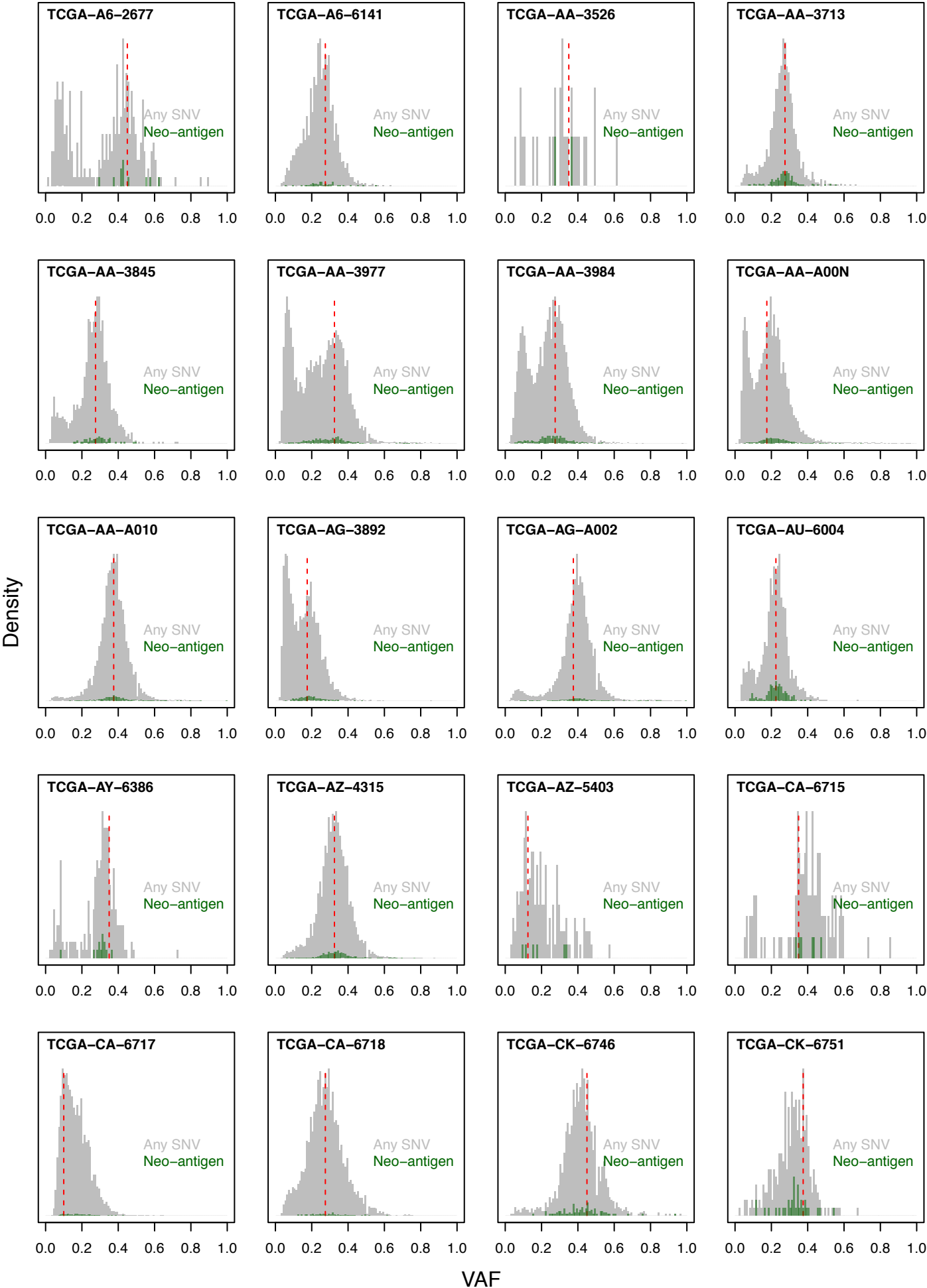

Supplement: Supplementary file 12 — Figure S11. Clonality of neoantigens in TCGA colorectal cancers Frequency histograms for cases corresponding to Figure 6 showing variant allele fraction (VAF) of all SNV mutations and predicted neo‐antigens. Only mutations in diploid regions of autosomes, and with coverage >20x were considered. Vertical red line indicates inferred clonal peak used to calculate cellularity. [file PATH-245-283-s011.pdf]
