# Supplementary material for: Somatic POLE exonuclease domain mutations are early events in sporadic endometrial and colorectal carcinogenesis, determining driver mutational landscape, clonal neoantigen burden and immune response
Source: J Pathol. 2018 Apr 30;245(3):283–96. doi: 10.1002/path.5081 (PMC6032922; doi:10.1002/path.5081)
Supplement: Supplementary file 13 — Table S1. Cohorts analysed and molecular analyses performed [file PATH-245-283-s013.docx]

**Table S1. Cohorts analysed and molecular analyses performed**

| **Cohort** | **Sample type** | **Analyses performed** | **Previous reports** |
| --- | --- | --- | --- |
| Paired endometrial cancers and EINs | FFPE tumour and paired precursor lesions | Molecular inversion probes capture panel sequencing  Custom Ion Torrent panel sequencing  Sanger sequencing  CD8 IHC | *POLE*-mutant endometrial cancers identified in [12, 14, 26] |
| Colorectal adenomas | FFPE adenomas | Sanger sequencing  Competitive allele-specific PCR (Taqman) | Previously unscreened |
| *POLE*-mutant endometrial and colorectal cancers | Fresh frozen tumours | Whole genome sequencing | *POLE* mutant invasive endometrial cancers from Leuven cohort identified in [12]. Other cases previously unreported. |
| TCGA endometrial cancers | Fresh frozen tumours | Re-analysis of genome and exome sequencing | TCGA [8] |
| TCGA colorectal cancers | Fresh frozen tumours | Re-analysis of genome and exome sequencing | TCGA [10] |
| Endometrial cancers | FFPE tumours | Thermo Comprehensive Cancer Panel | Cases identified in [14, 26] |

EIN – endometrial intraepithelial neoplasia

FFPE – formalin fixed paraffin embedded

IHC – immunohistochemistry
