# Supplementary material for: Somatic POLE exonuclease domain mutations are early events in sporadic endometrial and colorectal carcinogenesis, determining driver mutational landscape, clonal neoantigen burden and immune response
Source: J Pathol. 2018 Apr 30;245(3):283–96. doi: 10.1002/path.5081 (PMC6032922; doi:10.1002/path.5081)
Supplement: Supplementary file 14 — Table S2. Details of cases used for molecular analyses [file PATH-245-283-s014.docx]

**Table S2. Details of cases used for molecular analyses**

| **Case** | **Patient age** | **Sample type** | **Pathological characteristics** | **Tumour cell fraction** |
| --- | --- | --- | --- | --- |
| LUMC 33 | 61 | EIN |  | 60% |
|  |  | EEC | FIGO stage I, grade 2, LVSI neg | 70% |
| LUMC H0284 | 46 | EIN |  | 50% |
|  |  | EEC | FIGO stage III, grade 3, substantial LVSI | 70% |
| LUMC L2-18 | 49 | EIN |  | 70% |
|  |  | EEC | FIGO stage I, grade 1, LVSI neg | 70% |
| LUMC Q1-4 | 61 | EIN |  | 60% |
|  |  | EEC | FIGO stage I, grade 1, LVSI neg | 70% |
| OXF 001 | 55 | EEC | FIGO stage I, grade 2, LVSI neg | 70% |
| POLE 040 | 60 | Mixed EEC-serous | FIGO stage I, grade 3, LVSI neg | 60% |
| POLE 049 | 57 | Mixed-EEC-serous | FIGO stage I, grade 3, LVSI neg | 75% |
| POLE 072 | 55 | EEC | FIGO stage II, grade 3, LVSI neg | 70% |
| POLE 147 | 53 | EEC | FIGO stage I, grade 3, LVSI neg | 60% |
| BIR 001 | 61 | CRC | T4, N1 adenocarcinoma of descending colon | 75% |

EIN – endometrial intraepithelial neoplasia

EEC – endometrioid endometrial adenocarcinoma

FIGO – International Federation of Gynecology and Obstetrics

LVSI – lymphovascular space invasion
