# Supplementary material for: Somatic POLE exonuclease domain mutations are early events in sporadic endometrial and colorectal carcinogenesis, determining driver mutational landscape, clonal neoantigen burden and immune response
Source: J Pathol. 2018 Apr 30;245(3):283–96. doi: 10.1002/path.5081 (PMC6032922; doi:10.1002/path.5081)
Supplement: Supplementary file 15 — Table S3. Genes included in custom molecular inversion probe panel [file PATH-245-283-s015.docx]

**Table S3. Genes included in custom molecular inversion probe panel**

| **Gene** | **Region covered** |
| --- | --- |
| *ACVR2A* | Whole gene |
| *APC* | Codons 1–1600 |
| *ARID1A* | Whole gene |
| *ATM* | Whole gene |
| *B2M* | Whole gene |
| *BCL9L* | Whole gene |
| *BMPR2* | Whole gene |
| *BRAF* | Exons 11 and 15 |
| *CTNNB1* | Exons 1 to 3 |
| *ELF3* | Whole gene |
| *FBXW7* | Whole gene |
| *GNAS* | Codon 200 |
| *KRAS* | Whole gene |
| *MLH1* | Whole gene |
| *MSH2* | Whole gene |
| *MSH6* | Whole gene |
| *NRAS* | Whole gene |
| *PIK3CA* | Exons 9 and 20 |
| *POLD1* | Whole gene |
| *POLE* | Whole gene |
| *PTEN* | Whole gene |
| *RNF43* | Whole gene |
| *RPL22* | Whole gene |
| *SMAD2* | Whole gene |
| *SMAD4* | Whole gene |
| *SOX9* | Whole gene |
| *TCF7L2* | Whole gene |
| *TGIF1* | Whole gene |
| *TP53* | Whole gene |
| *ZFP36L2* | Whole gene |
