# Supplementary material for: Somatic POLE exonuclease domain mutations are early events in sporadic endometrial and colorectal carcinogenesis, determining driver mutational landscape, clonal neoantigen burden and immune response
Source: J Pathol. 2018 Apr 30;245(3):283–96. doi: 10.1002/path.5081 (PMC6032922; doi:10.1002/path.5081)
Supplement: Supplementary file 16 — Table S4. Genes included in custom Ion AmpliSeq Cancer Hotspot Panel [file PATH-245-283-s016.docx]

**Table S2. Genes included in custom Ion AmpliSeq Cancer Hotspot Panel**

| \| **Gene** \| **Region covered (chr: genomic region)** \| \| --- \| --- \| \| *ABL1* \| 9:133738295-133738378  9:133747443-133747530  9:133748280-133748417  9:133750308-133750405 \| \| *AKT1* \| 14:105241434-105241519  14:105246446-105246583 \| \| *ALK* \| 2:29432573-29432680  2:29443608-29443729 \| \| *AMELX* \| X:11315022-11315144 \| \| *AMELY* \| Y:6737844-6737961 \| \| *APC* \| 5:112173872-112173962  5:112174558-112174666  5:112175144-112175268  5:112175316-112175443  5:112175568-112175703  5:112175741-112175862  5:112175921-112176035 \| \| *ARAF* \| X:47422301-47422427  X:47424186-47424292  X:47424494-47424622  X:47426003-47426134  X:47426410-47426520  X:47426703-47426809  X:47428081-47428207  X:47430333-47430462  X:47430778-47430912 \| \| *ATM* \| 11:108117766-108117865  11:108119816-108119891  11:108123516-108123618  11:108137932-108138025  11:108155084-108155180  11:108170457-108170556  11:108172363-108172467  11:108173631-108173703  11:108180903-108180960  11:108200916-108200993  11:108204635-108204684  11:108205732-108205816  11:108206524-108206628  11:108218016-108218144  11:108225550-108225632  11:108236043-108236140  11:108236187-108236285 \| \| *BRAF* \| 7:140453103-140453221  7:140481392-140481515  7:140501290-140501404  7:140601429-140601547  7:140449769-140449884  7:140513594-140513702  7:140473222-140473348 \| \| *CARD11* \| 7:2979490-2979609 \| \| *CCND1* \| 11:69456121-69456217 \| \| *CD79A* \| 19:42384926-42385017 \| \| *CD79B* \| 17:62006543-62006654  17:62006745-62006851 \| \| *CDH1* \| 16:68835603-68835697  16:68846025-68846151  16:68847200-68847302 \| \| *CDK4* \| 12:58142281-58142408  12:58143003-58143109  12:58144601-58144720  12:58145099-58145223  12:58146676-58146776 \| \| *CDKN2A* \| 9:21970941-21971066  9:21971091-21971219  9:21968186-21968281  9:21973388-21973503  9:21940004-21940108  9:21975008-21975143  9:22029965-22030079  9:22024021-22024139  9:21968614-21968743  9:21971472-21971600  9:21961753-21961877 \| \| *CIC* \| 19:42791696-42791844 \| \| *CRNKL1* \| 20:20033041-20033157 \| \| *CSF1R* \| 5:149433594-149433692  5:149452992-149453073 \| \| *CTNNB1* \| 3:41241078-41241205  3:41265441-41265559  3:41266030-41266147  3:41266500-41266629  3:41268776-41268866  3:41274814-41274933  3:41277867-41277988  3:41280606-41280735 \| \| *DDX3Y* \| Y:15024738-15024861 \| \| *EGFR* \| 7:55211045-55211126  7:55221793-55221919  7:55232963-55233053  7:55241636-55241729  7:55242412-55242540  7:55248966-55249090  7:55249123-55249245  7:55259508-55259628  7:55135452-55135560  7:55306513-55306635  7:55193028-55193151  7:55223727-55223840  7:55223172-55223293  7:55101441-55101554 \| \| *EIF1AX* \| X:20148636-20148752  X:20150247-20150364  X:20152044-20152163  X:20153862-20153932  X:20159682-20159787 \| \| *ERBB2* \| 17:37868129-37868243  17:37880213-37880340  17:37880954-37881061  17:37881325-37881453  17:37865901-37866026  17:37853077-37853196  17:37843447-37843573  17:37879706-37879836 \| \| *ERBB3* \| 12:56492600-56492694 \| \| *ERBB4* \| 2:212288905-212288990  2:212530052-212530180  2:212576800-212576910  2:212578289-212578415  2:212587134-212587239  2:212589765-212589867  2:212652720-212652806  2:212812076-212812169 \| \| *EZH2* \| 7:148508707-148508791 \| \| *FBXW7* \| 4:153245411-153245492  4:153247278-153247369  4:153249356-153249477  4:153250853-153250926  4:153258902-153259023 \| \| *FGFR1* \| 8:38282141-38282254  8:38285852-38285975  8:38318251-38318370  8:38269418-38269545  8:38299680-38299806  8:38324508-38324628  8:38326396-38326506 \| \| *FGFR2* \| 10:123257953-123258045  10:123274722-123274835  10:123279418-123279544  10:123279608-123279713 \| \| *FGFR3* \| 4:1803552-1803653  4:1806082-1806187  4:1807834-1807930  4:1808312-1808399  4:1808882-1809006 \| \| *FLT3* \| 13:28592580-28592663  13:28602276-28602379  13:28608228-28608348  13:28610094-28610184 \| \| *FOXL2* \| 3:138664971-138665101  3:138665152-138665256  3:138665396-138665489 \| \| *GNA11* \| 19:3118882-3118973 \| \| *GNAQ* \| 9:80409376-80409498  9:80412437-80412553 \| \| *GNAS* \| 20:57484397-57484504  20:57484563-57484672 \| \| *H3F3A* \| 1:226252099-226252198 \| \| *H3F3B* \| 17:73775138-73775227 \| \| *HNF1A* \| 12:121431372-121431459  12:121432011-121432099 \| \| *HRAS* \| 11:533813-533930  11:534221-534308 \| \| *IDH1* \| 2:209113104-209113206 \| \| *IDH2* \| 15:90631825-90631954 \| \| *JAK2* \| 9:5073730-5073857 \| \| *JAK3* \| 19:17945617-17945734  19:17947987-17948074  19:17954135-17954225 \| \| *KDR* \| 4:55946089-55946208  4:55946251-55946371  4:55953776-55953860  4:55955079-55955168  4:55960977-55961059  4:55962445-55962548  4:55972953-55973071  4:55979575-55979655  4:55980239-55980359 \| \| *KIT* \| 4:55561655-55561784  4:55592158-55592246  4:55593418-55593513  4:55593576-55593695  4:55594171-55594279  4:55595497-55595562  4:55597437-55597524  4:55599281-55599358  4:55602674-55602751 \| \| *KNSTRN* \| 15:40675081-40675196 \| \| *KRAS* \| 12:25378550-25378658  12:25380261-25380364  12:25398187-25398304 \| \| *MAP2K1* \| 15:66727419-66727523  15:66729087-66729211  15:66735605-66735722  15:66774071-66774199  15:66777327-66777453  15:66782827-66782955 \| \| *MAP2K2* \| 19:4110512-4110641  19:4117464-4117567 \| \| *MAP2K4* \| 17:11958220-11958313  17:11998819-11998943  17:12011124-12011216  17:12032427-12032543 \| \| *MAP3K1* \| 5:56161134-56161241  5:56168493-56168590  5:56177793-56177914  5:56181669-56181783  5:56189300-56189423 \| \| *MDM2* \| 12:69207354-69207447  12:69210630-69210747  12:69214589-69214704  12:69218127-69218249  12:69229560-69229677  12:69230401-69230511 \| \| *MED12* \| X:70339230-70339346 \| \| *MET* \| 7:116339616-116339701  7:116340156-116340270  7:116403132-116403251  7:116411879-116411997  7:116417428-116417542  7:116423408-116423492  7:116408006-116408123  7:116354486-116354603  7:116370235-116370355  7:116378980-116379104  7:116321030-116321136  7:116437402-116437518  7:116457318-116457438  7:116225684-116225803  7:116226388-116226517 \| \| *MLH1* \| 3:37067208-37067333 \| \| *MPL* \| 1:43814969-43815086 \| \| *MUTYH* \| 1:45797149-45797271  1:45798403-45798526 \| \| *MYC* \| 8:128748451-128748582  8:128748850-128748926  8:128750132-128750265  8:128751051-128751178  8:128751804-128751916  8:128752915-128753030 \| \| *MYD88* \| 3:38181920-38182047  3:38182551-38182654 \| \| *NOTCH1* \| 9:139390765-139390885  9:139397763-139397879  9:139399338-139399447 \| \| *NPM1* \| 5:170837502-170837616 \| \| *NRAS* \| 1:115252186-115252269  1:115256505-115256584  1:115258690-115258774 \| \| *PDGFRA* \| 4:55140982-55141103  4:55144102-55144195  4:55144539-55144653  4:55152025-55152154 \| \| *PDGFRB* \| 5:149503733-149503865  5:149505087-149505194 \| \| *PIK3CA* \| 3:178916776-178916881  3:178916932-178917035  3:178921465-178921570  3:178927406-178927525  3:178927902-178927986  3:178928070-178928160  3:178936024-178936105  3:178938788-178938918  3:178947819-178947896  3:178951997-178952097  3:178952141-178952237 \| \| *POLE* \| 12:133249778-133249897  12:133250221-133250314  12:133252000-133252107  12:133252298-133252426  12:133252672-133252797  12:133253119-133253245 \| \| *PPP2R1A* \| 19:52715904-52716031 \| \| *PTEN* \| 10:89624208-89624300  10:89685259-89685374  10:89692814-89692920  10:89711805-89711932  10:89717504-89717620  10:89717667-89717780  10:89720696-89720747  10:89720785-89720900 \| \| *PTK2* \| 8:141669617-141669742  8:141712703-141712817  8:141745421-141745529  8:141829004-141829113  8:141900745-141900841 \| \| *PTPN11* \| 12:112888119-112888228  12:112926836-112926961 \| \| *RB1* \| 13:48919224-48919312  13:48923140-48923255  13:48941602-48941724  13:48942598-48942711  13:48953754-48953874  13:48955526-48955605  13:49027106-49027178  13:49033828-49033934  13:49037847-49037932  13:49039150-49039232  13:49015985-49016102  13:48934473-48934591  13:48891745-48891867  13:48976663-48976783 \| \| *RET* \| 10:43609067-43609182  10:43609875-43610010  10:43613795-43613893  10:43615547-43615687  10:43617315-43617433 \| \| *SMAD4* \| 18:48575100-48575213  18:48575557-48575677  18:48581191-48581302  18:48584552-48584678  18:48586252-48586361  18:48591815-48591931  18:48593400-48593519  18:48603029-48603119  18:48604659-48604774 \| \| *SMARCB1* \| 22:24133954-24134064  22:24143201-24143311  22:24145478-24145598  22:24176260-24176391 \| \| *SMO* \| 7:128845064-128845188  7:128845958-128846063  7:128846338-128846419  7:128850270-128850363  7:128851500-128851612 \| \| *SRC* \| 20:36031667-36031769 \| \| *STK11* \| 19:1206978-1207104  19:1220311-1220450  19:1220481-1220603  19:1221237-1221332  19:1223015-1223144 \| \| *TP53* \| 17:7573924-7574035  17:7576495-7576597  17:7576799-7576924  17:7577016-7577151  17:7577509-7577612  17:7578181-7578298  17:7578353-7578483  17:7578517-7578601  17:7579351-7579485  17:7579610-7579735  17:7579854-7579960  17:7560788-7560911  17:7606938-7607064  17:7571397-7571521  17:7553689-7553811  17:7554704-7554830  17:7557749-7557878  17:7592084-7592200  17:7589231-7589350 \| \| *VHL* \| 3:10183766-10183854  3:10188187-10188306  3:10191419-10191527 \| \|  \| 1:42844194-42844315  2:60805293-60805413  3:78775495-78775594  5:172960747-172960867  6:82610964-82611093  7:121653293-121653411  8:102550754-102550876  9:23132146-23132272  11:29385066-29385177  13:34601802-34601922  14:27326888-27327009  14:37148868-37148991  15:101111848-101111970  17:14400867-14400943  18:7751698-7751818  18:62023770-62023888  16:66009899-66010026  19:44300771-44300896  19:57764725-57764847  20:39521510-39521639  20:3964993-3965117  21:30365226-30365353  22:20220682-20220806 \| |  |
| --- | --- | --- | --- | --- | --- | --- | --- | --- | --- | --- | --- | --- | --- | --- | --- | --- | --- | --- | --- | --- | --- | --- | --- | --- | --- | --- | --- | --- | --- | --- | --- | --- | --- | --- | --- | --- | --- | --- | --- | --- | --- | --- | --- | --- | --- | --- | --- | --- | --- | --- | --- | --- | --- | --- | --- | --- | --- | --- | --- | --- | --- | --- | --- | --- | --- | --- | --- | --- | --- | --- | --- | --- | --- | --- | --- | --- | --- | --- | --- | --- | --- | --- | --- | --- | --- | --- | --- | --- | --- | --- | --- | --- | --- | --- | --- | --- | --- | --- | --- | --- | --- | --- | --- | --- | --- | --- | --- | --- | --- | --- | --- | --- | --- | --- | --- | --- | --- | --- | --- | --- | --- | --- | --- | --- | --- | --- | --- | --- | --- | --- | --- | --- | --- | --- | --- | --- | --- | --- | --- | --- | --- | --- | --- | --- | --- | --- | --- | --- | --- | --- | --- | --- | --- | --- | --- | --- | --- | --- | --- | --- | --- | --- | --- | --- | --- |
