# Supplementary material for: Somatic POLE exonuclease domain mutations are early events in sporadic endometrial and colorectal carcinogenesis, determining driver mutational landscape, clonal neoantigen burden and immune response
Source: J Pathol. 2018 Apr 30;245(3):283–96. doi: 10.1002/path.5081 (PMC6032922; doi:10.1002/path.5081)
Supplement: Supplementary file 17 — Table S5. List of IntOGen endometrial cancer driver genes used in this study [file PATH-245-283-s017.docx]

**Table S5. List of IntOGen endometrial cancer driver genes used in this study**

| **No.** | **HUGO symbol** |
| --- | --- |
| 1 | ACACA |
| 2 | ACTB |
| 3 | ACTG1 |
| 4 | AHR |
| 5 | AKT1 |
| 6 | ALK |
| 7 | ANK3 |
| 8 | APC |
| 9 | ARAP3 |
| 10 | ARHGAP35 |
| 11 | ARHGEF6 |
| 12 | ARID1A |
| 13 | ARID5B |
| 14 | ARNTL |
| 15 | ATF1 |
| 16 | ATIC |
| 17 | ATM |
| 18 | ATR |
| 19 | AXIN1 |
| 20 | BAZ2B |
| 21 | BCLAF1 |
| 22 | BMPR2 |
| 23 | BRAF |
| 24 | BRCA1 |
| 25 | CAPN7 |
| 26 | CARM1 |
| 27 | CAST |
| 28 | CAT |
| 29 | CCND1 |
| 30 | CDKN1B |
| 31 | CHD3 |
| 32 | CHD4 |
| 33 | CHD9 |
| 34 | CHEK2 |
| 35 | CLOCK |
| 36 | CLTC |
| 37 | CNOT4 |
| 38 | CSNK1G3 |
| 39 | CTCF |
| 40 | CTNNB1 |
| 41 | CTNND1 |
| 42 | CUL1 |
| 43 | CUX1 |
| 44 | DEPDC1B |
| 45 | DHX15 |
| 46 | DHX35 |
| 47 | DICER1 |
| 48 | DIS3 |
| 49 | DNMT3A |
| 50 | EGFR |
| 51 | EIF1AX |
| 52 | EIF2AK3 |
| 53 | EIF2C3 |
| 54 | EIF4A2 |
| 55 | EIF4G1 |
| 56 | EP300 |
| 57 | ERBB3 |
| 58 | FAM123B |
| 59 | FAS |
| 60 | FBXW7 |
| 61 | FGFR2 |
| 62 | FLT3 |
| 63 | FOXA2 |
| 64 | FUBP1 |
| 65 | FXR1 |
| 66 | G3BP2 |
| 67 | GNAI1 |
| 68 | GPS2 |
| 69 | GPSM2 |
| 70 | HDAC3 |
| 71 | HGF |
| 72 | IDH1 |
| 73 | ING1 |
| 74 | INPP4A |
| 75 | INPPL1 |
| 76 | IREB2 |
| 77 | KDM6A |
| 78 | KLF4 |
| 79 | KRAS |
| 80 | MAP2K4 |
| 81 | MAP3K1 |
| 82 | MAX |
| 83 | MED17 |
| 84 | MET |
| 85 | MGA |
| 86 | MKL1 |
| 87 | MLH1 |
| 88 | MLH3 |
| 89 | MUC20 |
| 90 | MYB |
| 91 | MYH10 |
| 92 | NCF2 |
| 93 | NCKAP1 |
| 94 | NCOR1 |
| 95 | NDRG1 |
| 96 | NEDD4L |
| 97 | NF1 |
| 98 | NF2 |
| 99 | NFE2L2 |
| 100 | NR2F2 |
| 101 | NRAS |
| 102 | NUP93 |
| 103 | PCDH18 |
| 104 | PGR |
| 105 | PHF6 |
| 106 | PIK3CA |
| 107 | PIK3R1 |
| 108 | PIK3R3 |
| 109 | PLCG1 |
| 110 | PLXNB2 |
| 111 | POLE |
| 112 | PPP2R1A |
| 113 | PPP2R5A |
| 114 | PPP2R5C |
| 115 | PRPF8 |
| 116 | PRRX1 |
| 117 | PTEN |
| 118 | PTPN11 |
| 119 | RAD21 |
| 120 | RAD23B |
| 121 | RB1 |
| 122 | RBBP7 |
| 123 | RBM5 |
| 124 | RHEB |
| 125 | ROBO2 |
| 126 | RPL22 |
| 127 | RPL5 |
| 128 | RTN4 |
| 129 | RUNX1 |
| 130 | SEC31A |
| 131 | SHMT1 |
| 132 | SMAD2 |
| 133 | SMC1A |
| 134 | SOX17 |
| 135 | SPOP |
| 136 | SRGAP3 |
| 137 | STIP1 |
| 138 | SUZ12 |
| 139 | SYNCRIP |
| 140 | TBL1XR1 |
| 141 | TBX3 |
| 142 | TFDP1 |
| 143 | TGFBR2 |
| 144 | TP53 |
| 145 | TP53BP1 |
| 146 | U2AF1 |
| 147 | VHL |
| 148 | WIPF1 |
| 149 | ZC3H11A |
| 150 | ZFHX3 |
| 151 | ZFP36L2 |
| 152 | ZMYM2 |
| 153 | ZNF814 |
