# Supplementary material for: Somatic POLE exonuclease domain mutations are early events in sporadic endometrial and colorectal carcinogenesis, determining driver mutational landscape, clonal neoantigen burden and immune response
Source: J Pathol. 2018 Apr 30;245(3):283–96. doi: 10.1002/path.5081 (PMC6032922; doi:10.1002/path.5081)
Supplement: Supplementary file 18 — Table S6. List of IntOGen colorectal cancer driver genes used in this study [file PATH-245-283-s018.docx]

**Table S6. List of IntOGen colorectal cancer driver genes used in this study**

| **No.** | **HUGO symbol** |
| --- | --- |
| 1 | ACO1 |
| 2 | ACSL6 |
| 3 | ACVR1B |
| 4 | AKAP9 |
| 5 | APC |
| 6 | ARID1A |
| 7 | ARNTL |
| 8 | ASPM |
| 9 | ATM |
| 10 | ATRX |
| 11 | AXIN2 |
| 12 | BCOR |
| 13 | BMPR2 |
| 14 | BPTF |
| 15 | BRAF |
| 16 | BRWD1 |
| 17 | CAD |
| 18 | CASP8 |
| 19 | CDC73 |
| 20 | CDK12 |
| 21 | CDKN1B |
| 22 | CEP290 |
| 23 | CHD4 |
| 24 | CHD9 |
| 25 | CLSPN |
| 26 | CNOT1 |
| 27 | CREBBP |
| 28 | CTCF |
| 29 | CTNNB1 |
| 30 | CUL1 |
| 31 | DIS3 |
| 32 | DNMT3A |
| 33 | EGFR |
| 34 | ELF3 |
| 35 | FAM123B |
| 36 | FBXW7 |
| 37 | FN1 |
| 38 | FOXP1 |
| 39 | FXR1 |
| 40 | GATA3 |
| 41 | GNAS |
| 42 | GOLGA5 |
| 43 | IDH2 |
| 44 | ITSN1 |
| 45 | KRAS |
| 46 | LPHN2 |
| 47 | MAP2K1 |
| 48 | MAP3K4 |
| 49 | MECOM |
| 50 | MED12 |
| 51 | MED24 |
| 52 | MGA |
| 53 | MLL2 |
| 54 | MSR1 |
| 55 | MYH10 |
| 56 | NF1 |
| 57 | NR2F2 |
| 58 | NR4A2 |
| 59 | NRAS |
| 60 | NTN4 |
| 61 | NUP107 |
| 62 | NUP98 |
| 63 | PCBP1 |
| 64 | PIK3CA |
| 65 | PIK3R1 |
| 66 | POLR2B |
| 67 | PPP2R1A |
| 68 | PTEN |
| 69 | PTGS1 |
| 70 | PTPN11 |
| 71 | PTPRU |
| 72 | RAD21 |
| 73 | RBM10 |
| 74 | RTN4 |
| 75 | RUNX1 |
| 76 | SF3B1 |
| 77 | SMAD2 |
| 78 | SMAD4 |
| 79 | SMC1A |
| 80 | SOS2 |
| 81 | SOX9 |
| 82 | SRGAP3 |
| 83 | STAG2 |
| 84 | SYNCRIP |
| 85 | TAF1 |
| 86 | TBX3 |
| 87 | TCF12 |
| 88 | TCF7L2 |
| 89 | TGFBR2 |
| 90 | TP53 |
| 91 | TP53BP1 |
| 92 | TRIO |
| 93 | WIPF1 |
| 94 | WT1 |
| 95 | ZC3H11A |
| 96 | ACO1 |
